# Supplementary figures and images for: A bacterial gene acquired by parasitoid wasps contributes to venom secretion against host defence
Source: EMBO J. 2026 Jan 28;45(6):1997–2029. doi: 10.1038/s44318-026-00702-6 (PMC12992598; doi:10.1038/s44318-026-00702-6)

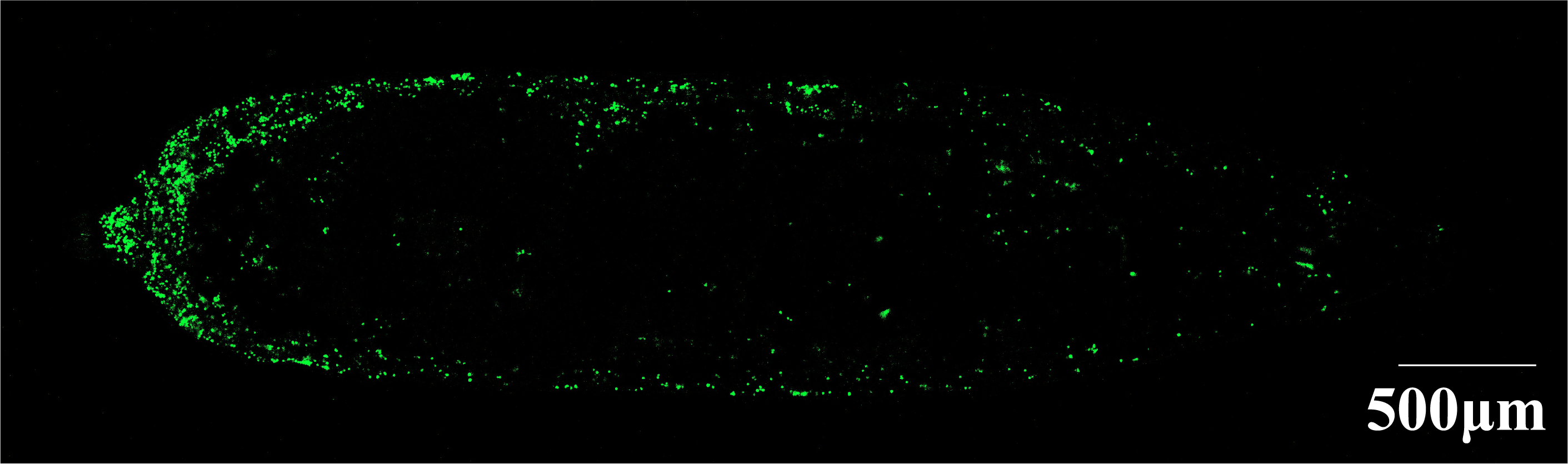

Supplement: Supplementary file 19 — Source data Fig. 2 [file 44318_2026_702_MOESM19_ESM.zip › Figure 2/2C/host larvae parasitized by L. heterotoma (dsGFP).tif]

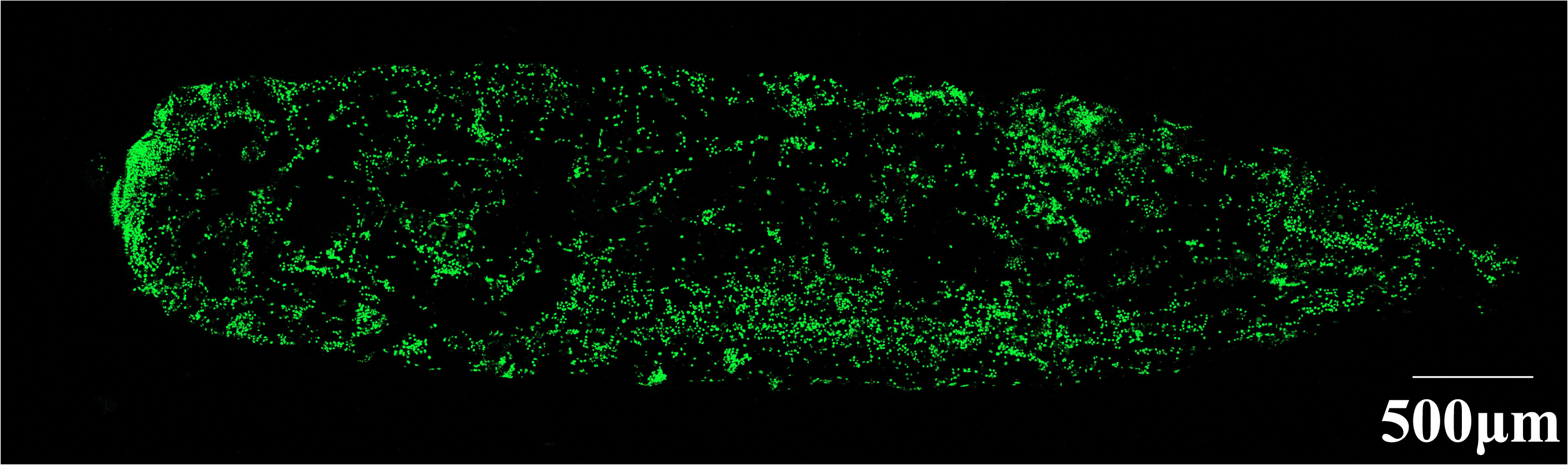

Supplement: Supplementary file 19 — Source data Fig. 2 [file 44318_2026_702_MOESM19_ESM.zip › Figure 2/2C/host larvae parasitized by L. heterotoma (dsPTCDS).tif]

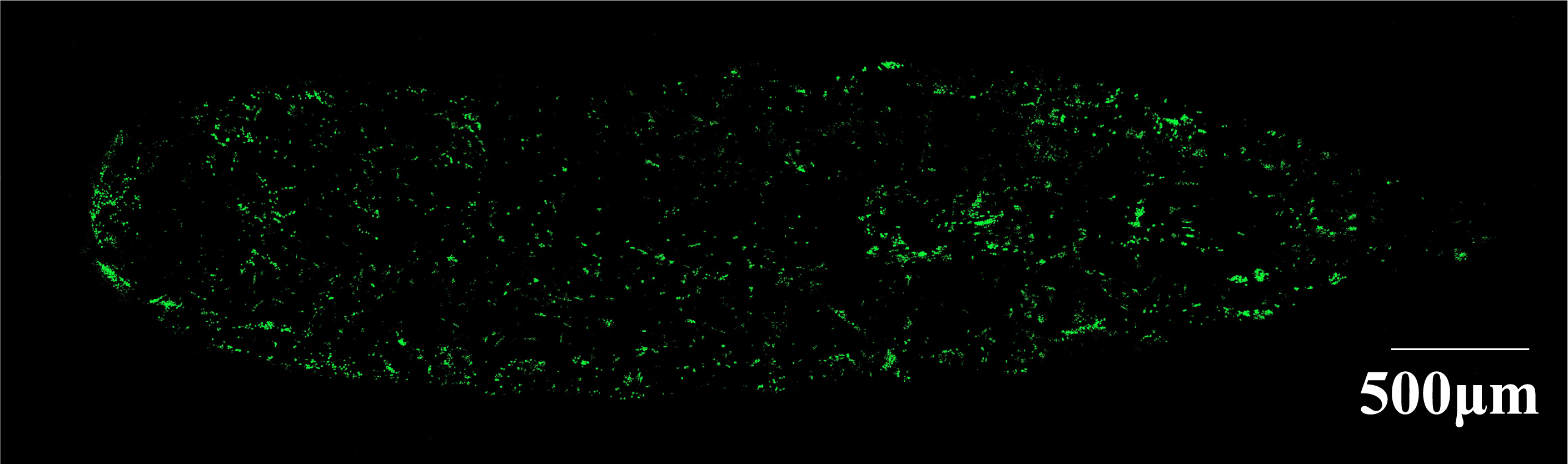

Supplement: Supplementary file 19 — Source data Fig. 2 [file 44318_2026_702_MOESM19_ESM.zip › Figure 2/2C/host larvae parasitized by L. syphax (dsGFP).tif]

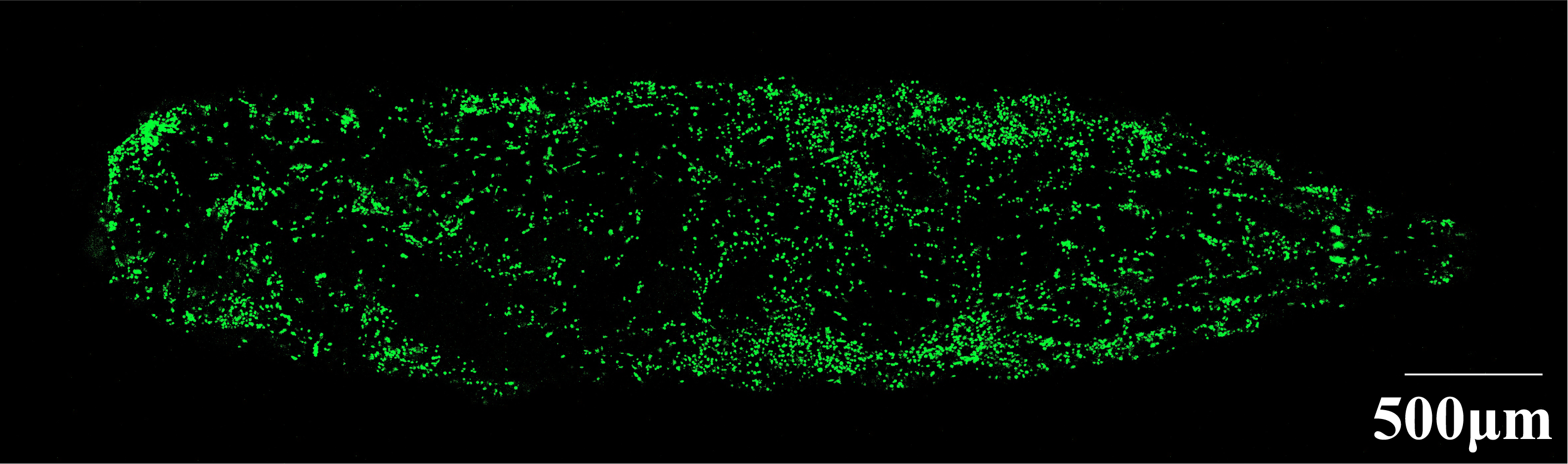

Supplement: Supplementary file 19 — Source data Fig. 2 [file 44318_2026_702_MOESM19_ESM.zip › Figure 2/2C/host larvae parasitized by L. syphax (dsPTCDS).tif]

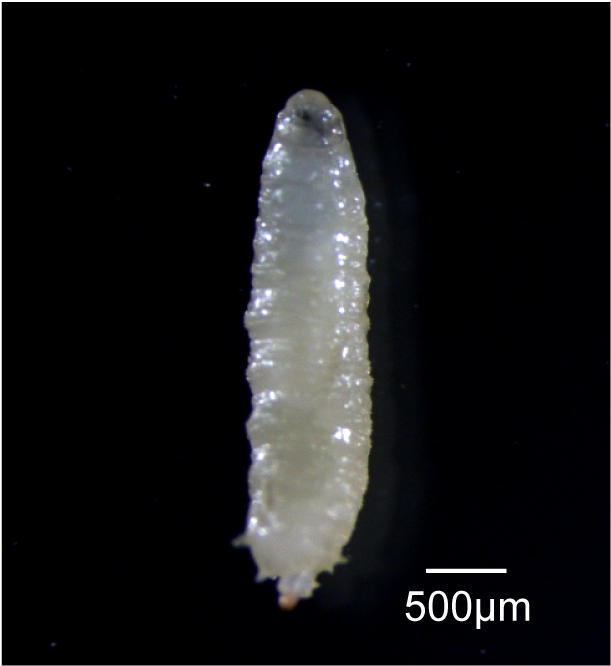

Supplement: Supplementary file 19 — Source data Fig. 2 [file 44318_2026_702_MOESM19_ESM.zip › Figure 2/2B/host larvae parasitized by L. heterotoma (dsGFP).tif]

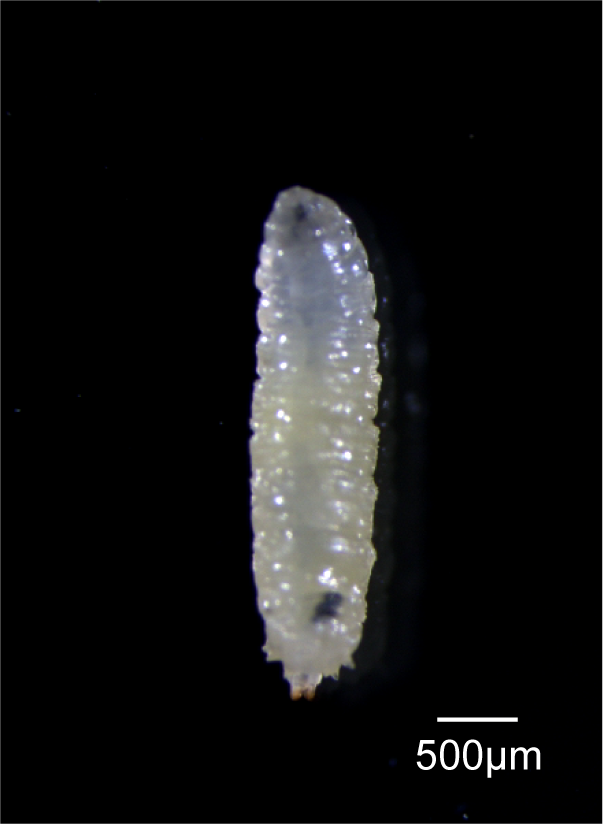

Supplement: Supplementary file 19 — Source data Fig. 2 [file 44318_2026_702_MOESM19_ESM.zip › Figure 2/2B/host larvae parasitized by L. heterotoma (dsPTCDS).tif]

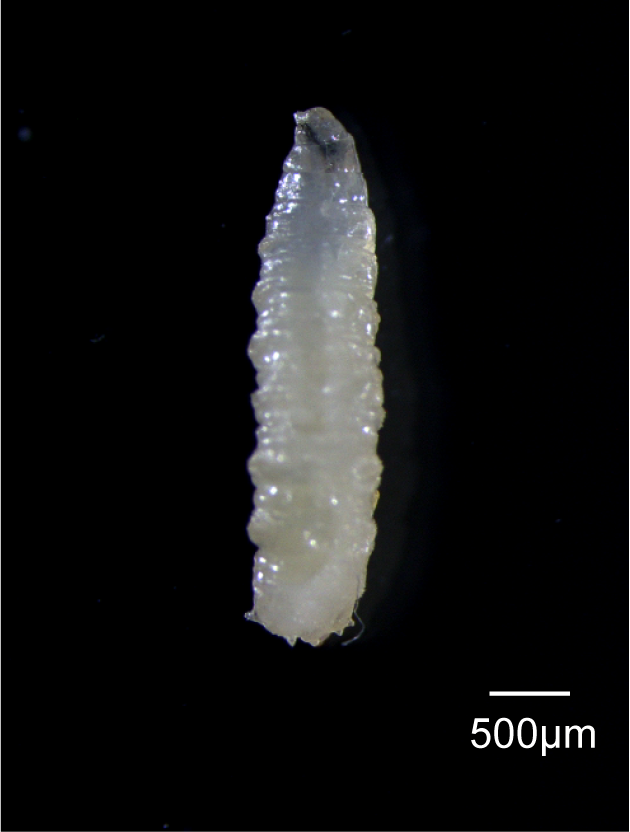

Supplement: Supplementary file 19 — Source data Fig. 2 [file 44318_2026_702_MOESM19_ESM.zip › Figure 2/2B/host larvae parasitized by L. syphax (dsGFP).tif]

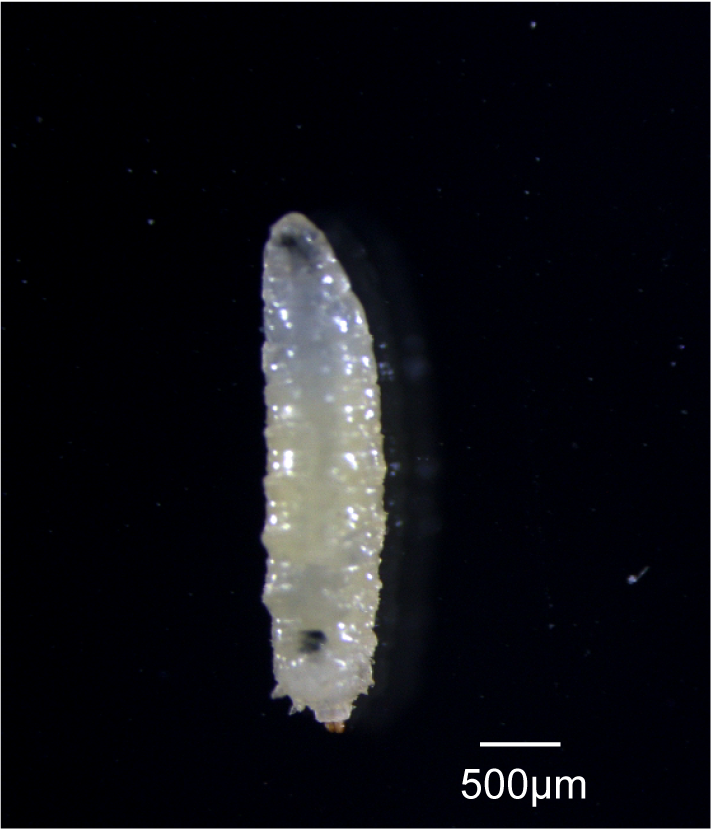

Supplement: Supplementary file 19 — Source data Fig. 2 [file 44318_2026_702_MOESM19_ESM.zip › Figure 2/2B/host larvae parasitized by L. syphax (dsPTCDS).tif]

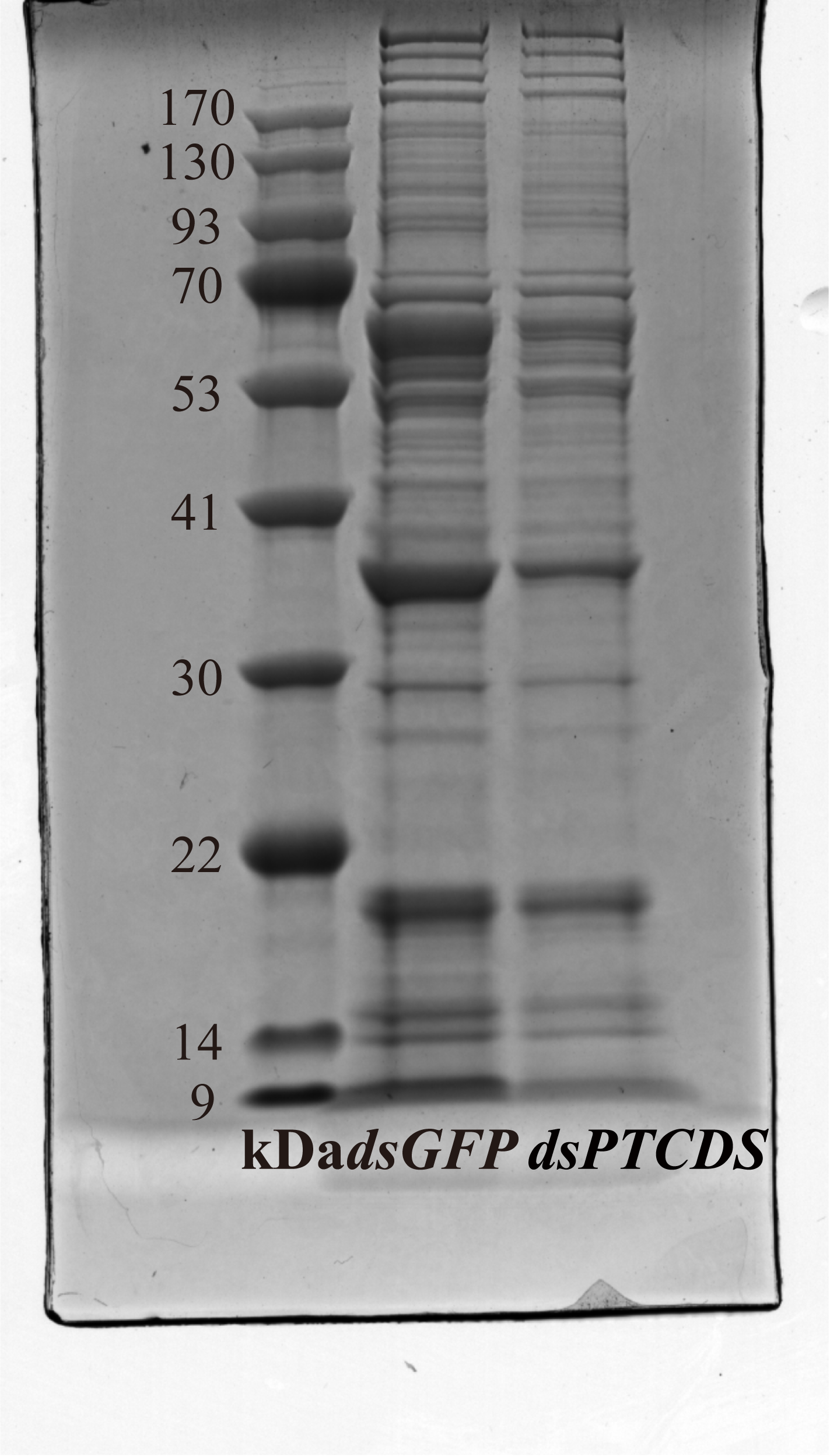

Supplement: Supplementary file 20 — Source data Fig. 3 [file 44318_2026_702_MOESM20_ESM.zip › Figure 3/3B/SDS-PAGE gel electrophoretic profile.tif]

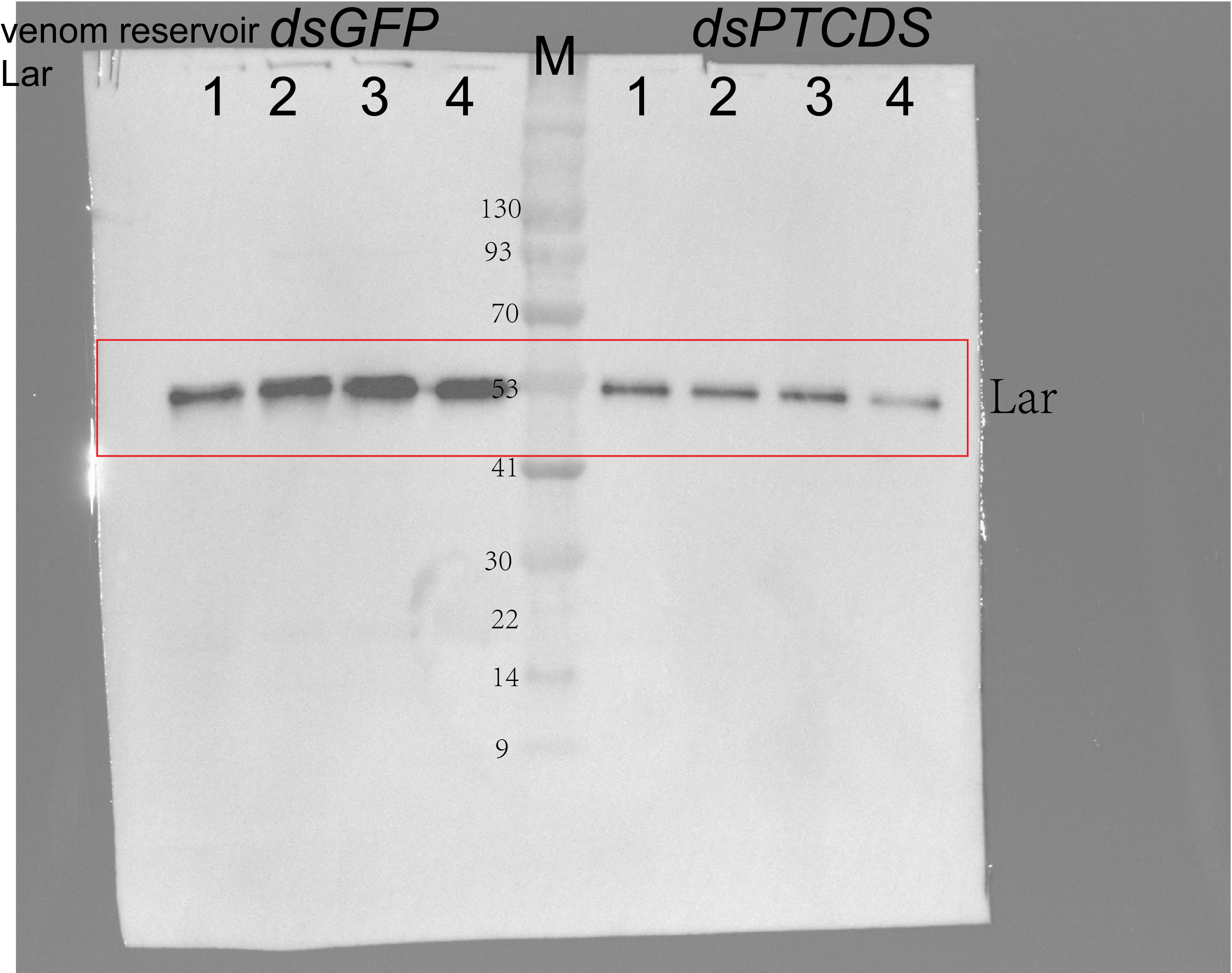

Supplement: Supplementary file 20 — Source data Fig. 3 [file 44318_2026_702_MOESM20_ESM.zip › Figure 3/3C/western blot-Lar (venom reservoir).tif]

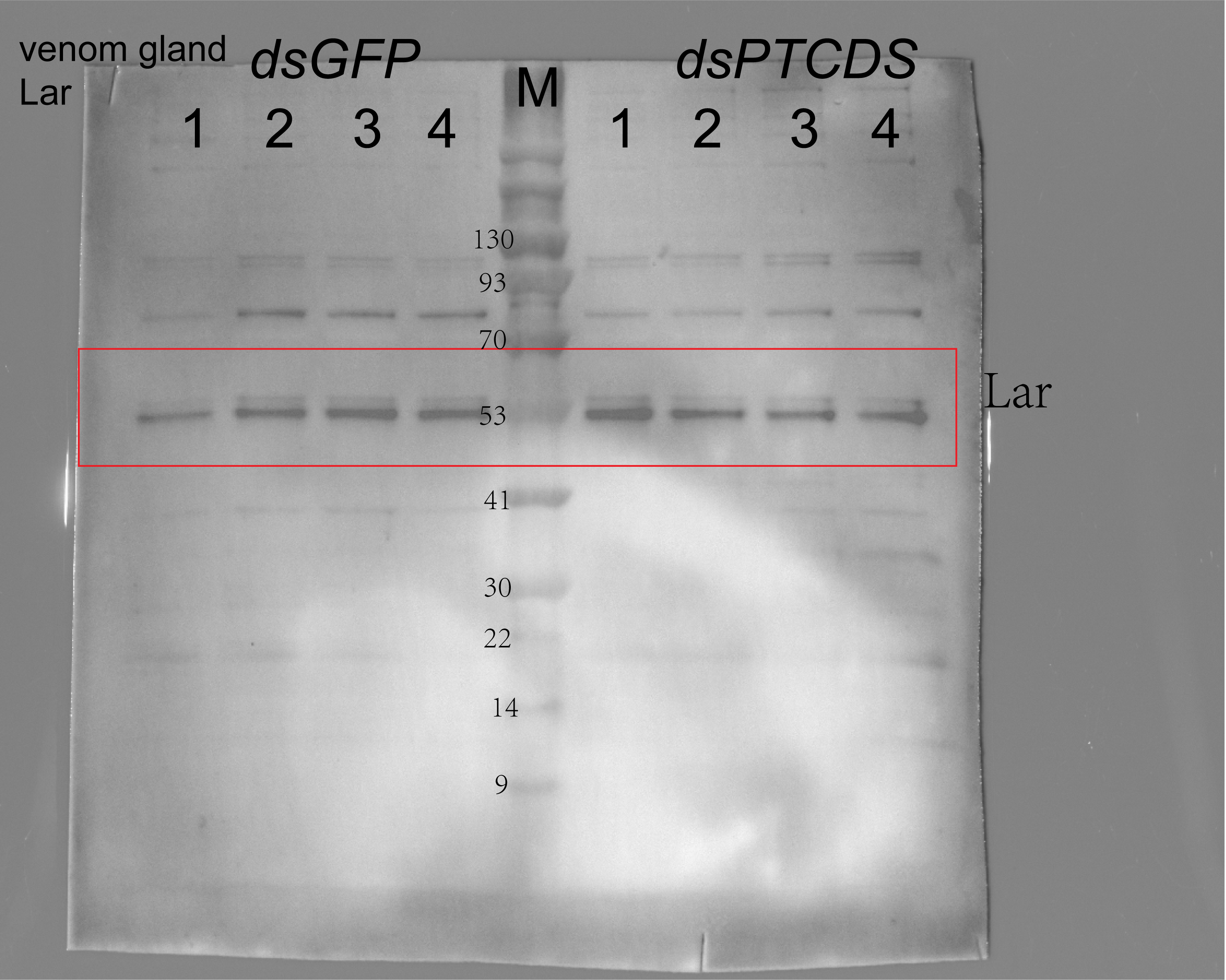

Supplement: Supplementary file 20 — Source data Fig. 3 [file 44318_2026_702_MOESM20_ESM.zip › Figure 3/3C/western blot-Lar (venom gland).tif]

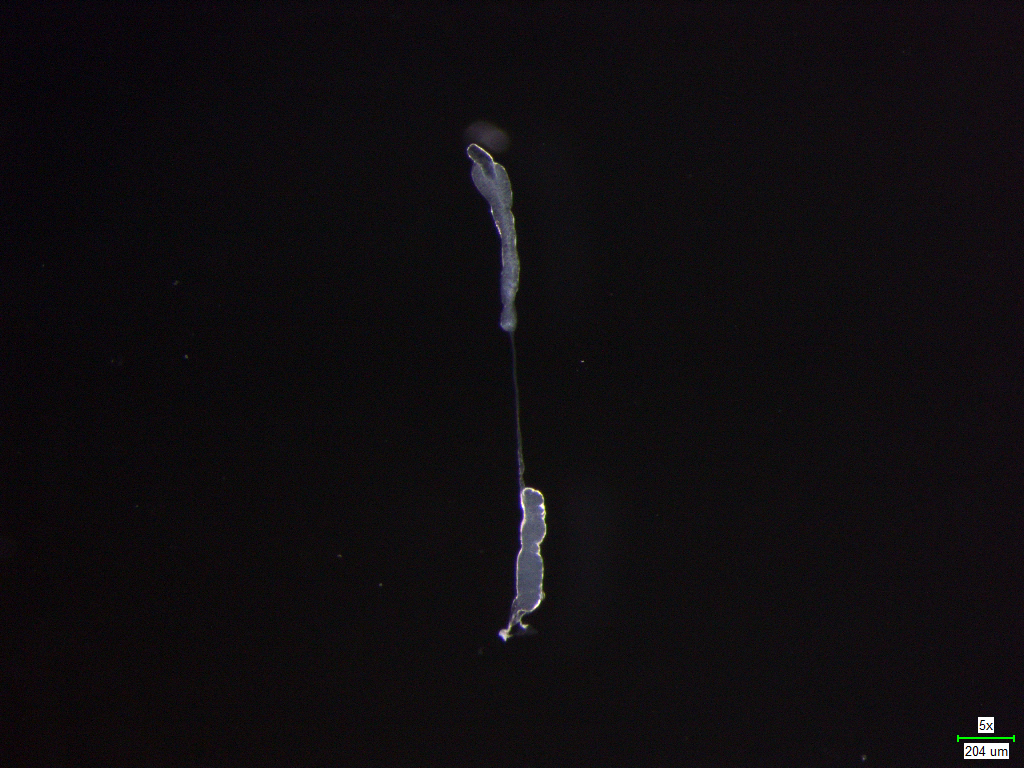

Supplement: Supplementary file 20 — Source data Fig. 3 [file 44318_2026_702_MOESM20_ESM.zip › Figure 3/3A/venom gland and venom reservoir in L. heterotoma (dsPTCDS).tif]

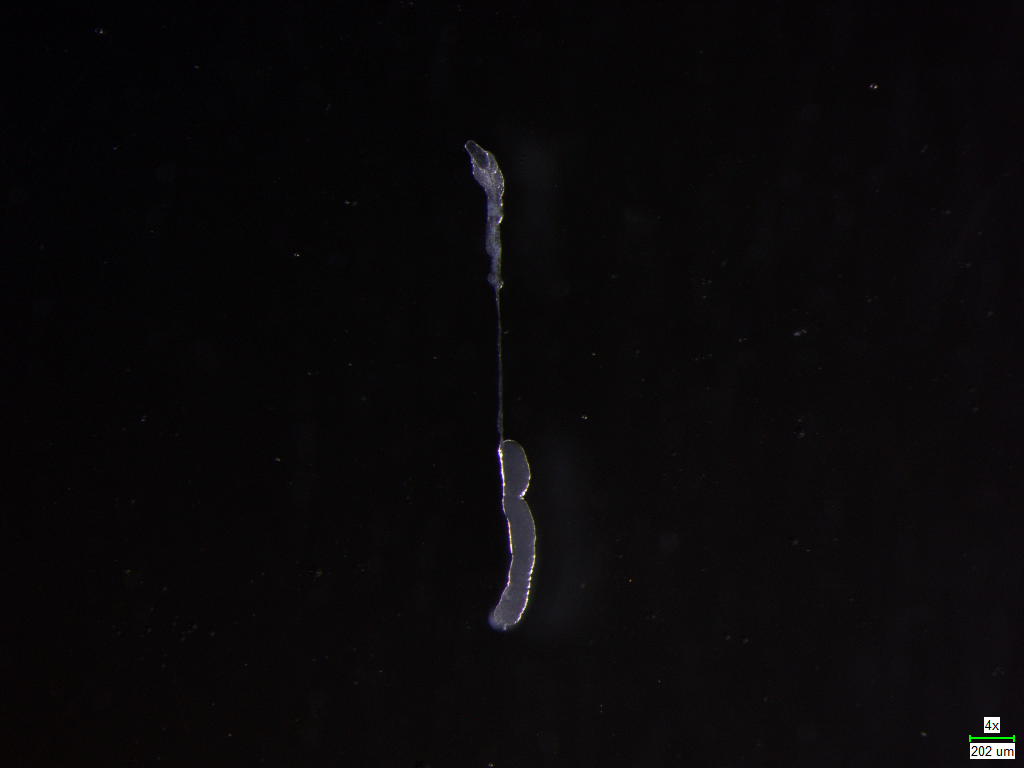

Supplement: Supplementary file 20 — Source data Fig. 3 [file 44318_2026_702_MOESM20_ESM.zip › Figure 3/3A/venom gland and venom reservoir in L. heterotoma (dsGFP).tif]

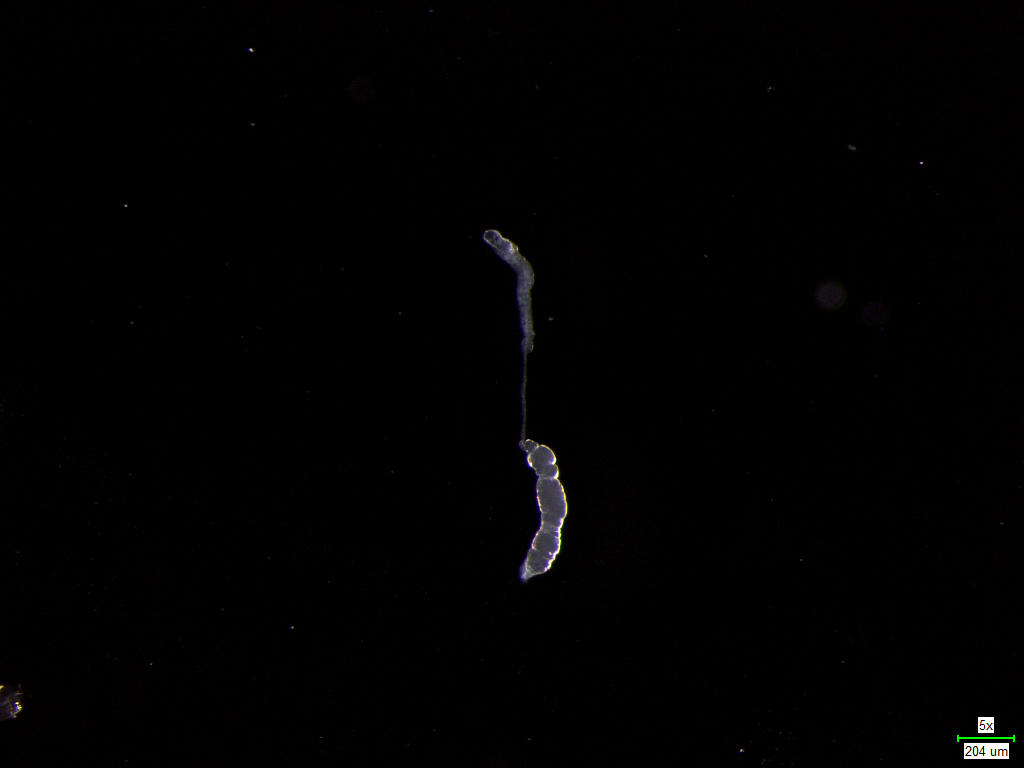

Supplement: Supplementary file 20 — Source data Fig. 3 [file 44318_2026_702_MOESM20_ESM.zip › Figure 3/3A/venom gland and venom reservoir in L. syphax (dsGFP).tif]

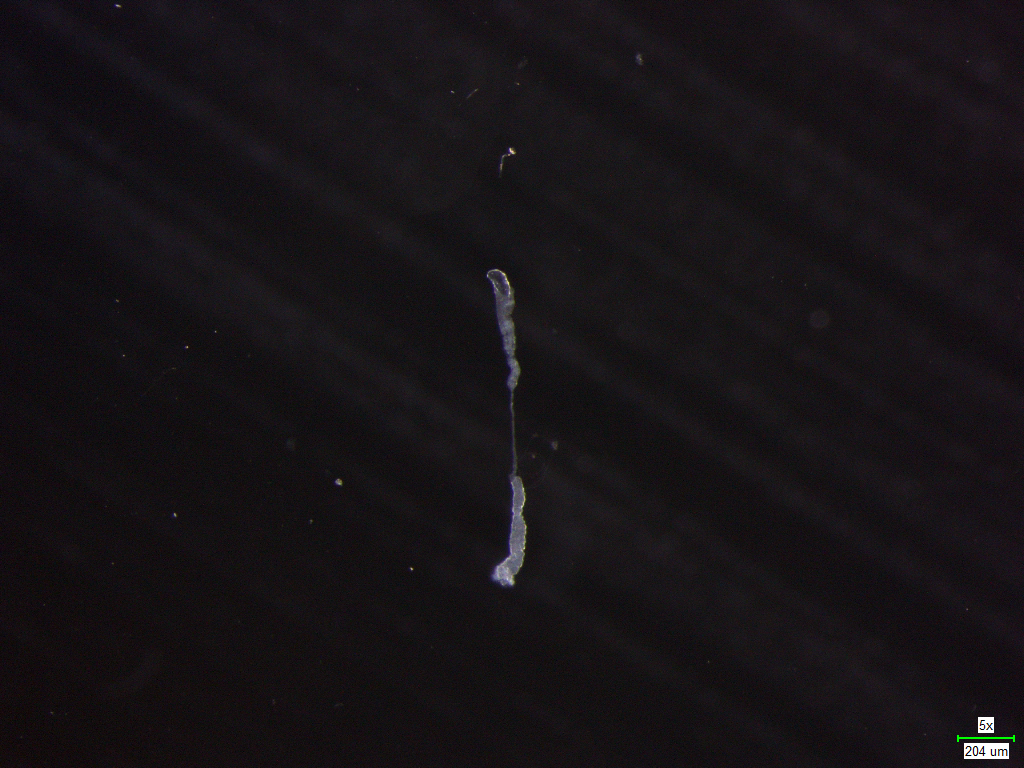

Supplement: Supplementary file 20 — Source data Fig. 3 [file 44318_2026_702_MOESM20_ESM.zip › Figure 3/3A/venom gland and venom reservoir in L. syphax (dsPTCDS).tif]

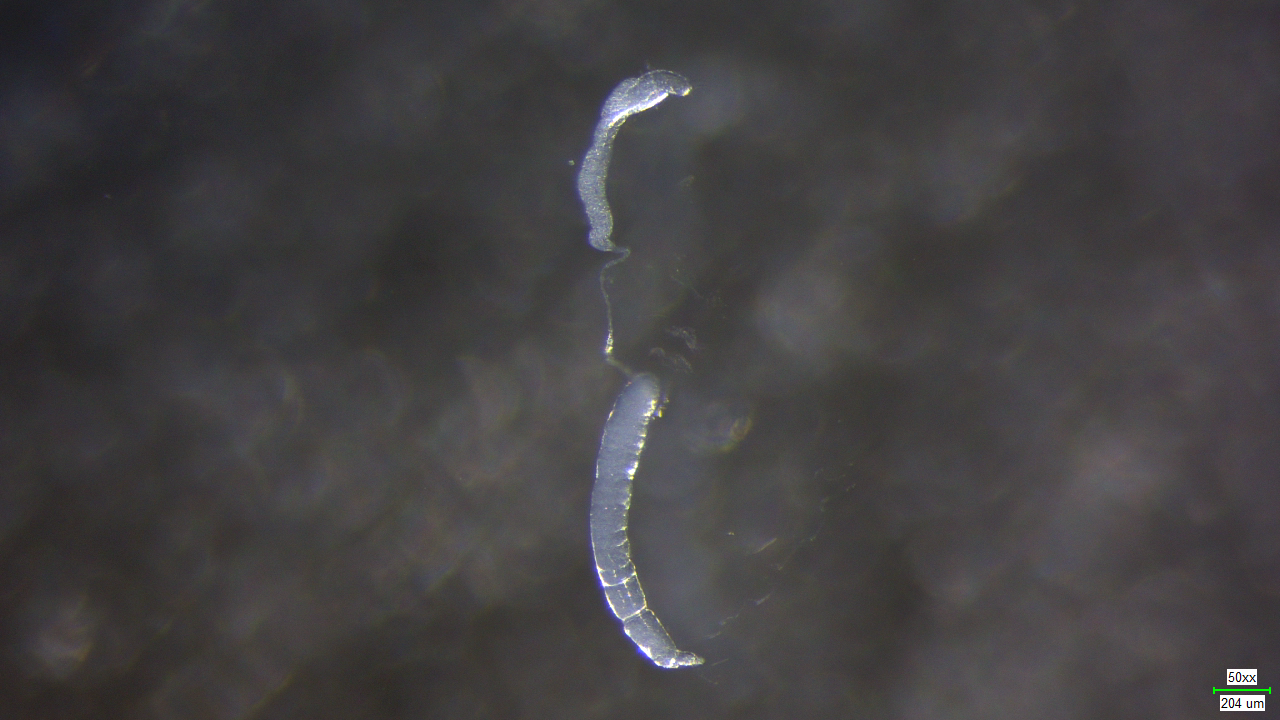

Supplement: Supplementary file 21 — Source data Fig. 4 [file 44318_2026_702_MOESM21_ESM.zip › Figure 4/4D/dsGFP.tif]

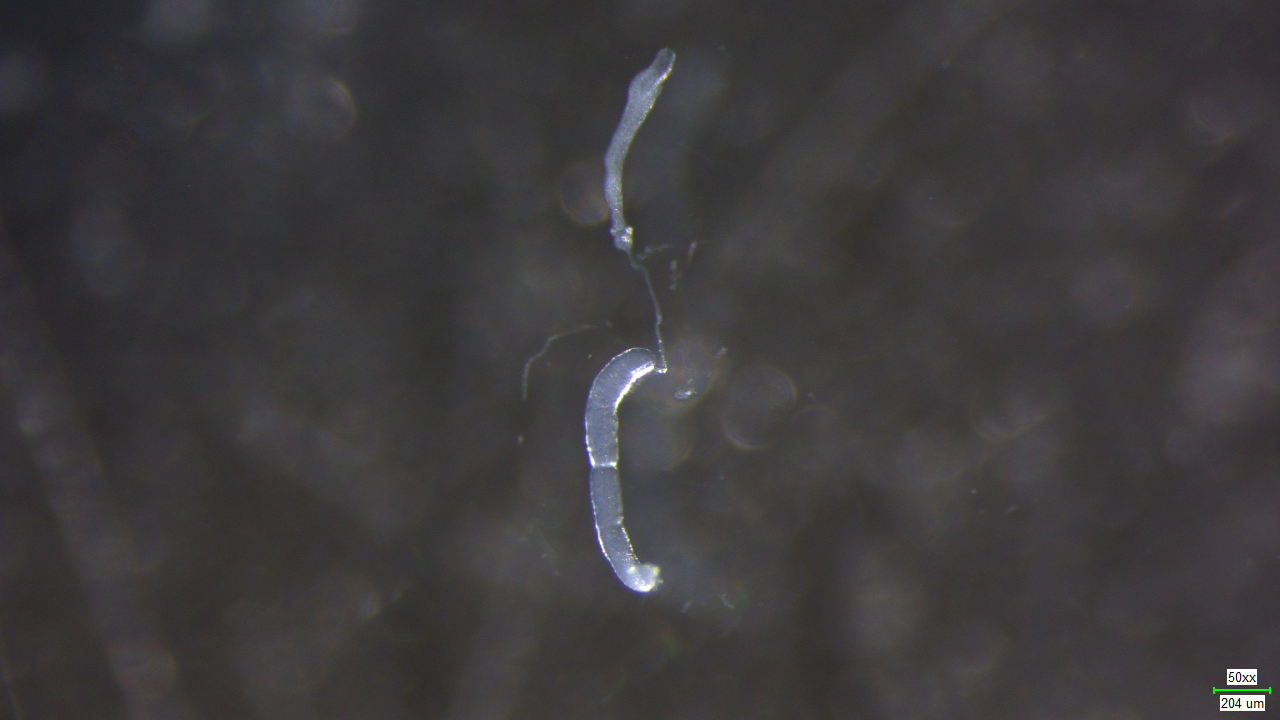

Supplement: Supplementary file 21 — Source data Fig. 4 [file 44318_2026_702_MOESM21_ESM.zip › Figure 4/4D/dsSar1.tif]

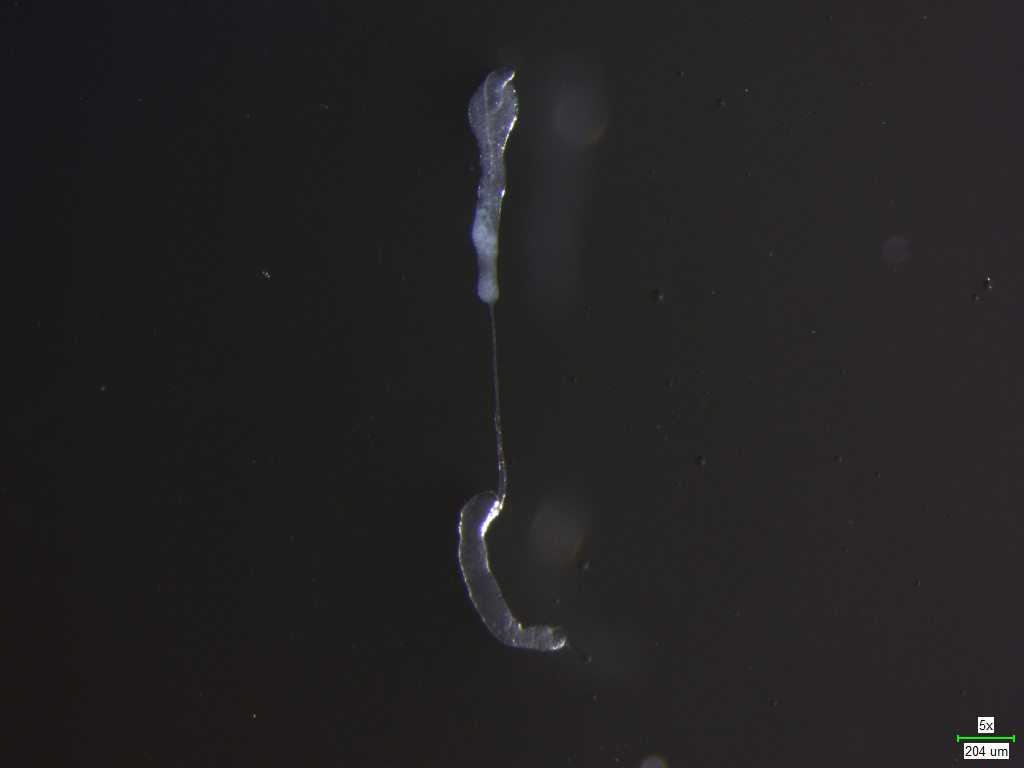

Supplement: Supplementary file 21 — Source data Fig. 4 [file 44318_2026_702_MOESM21_ESM.zip › Figure 4/4D/dsCHOp24.tif]

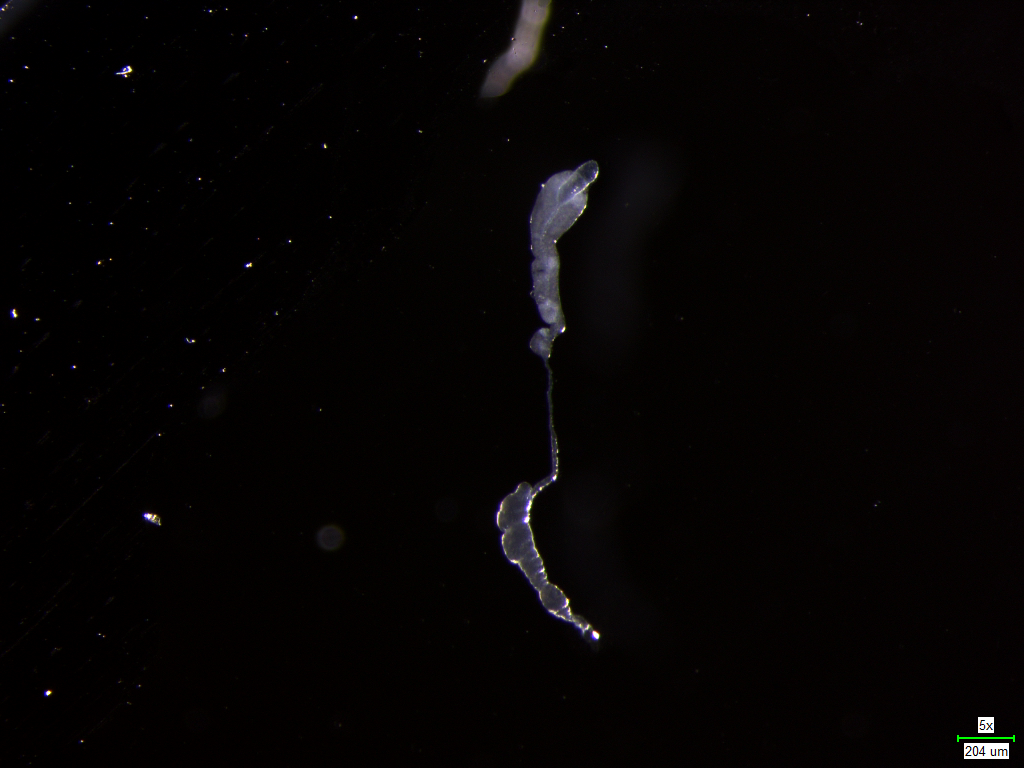

Supplement: Supplementary file 21 — Source data Fig. 4 [file 44318_2026_702_MOESM21_ESM.zip › Figure 4/4D/dsP24-1.tif]

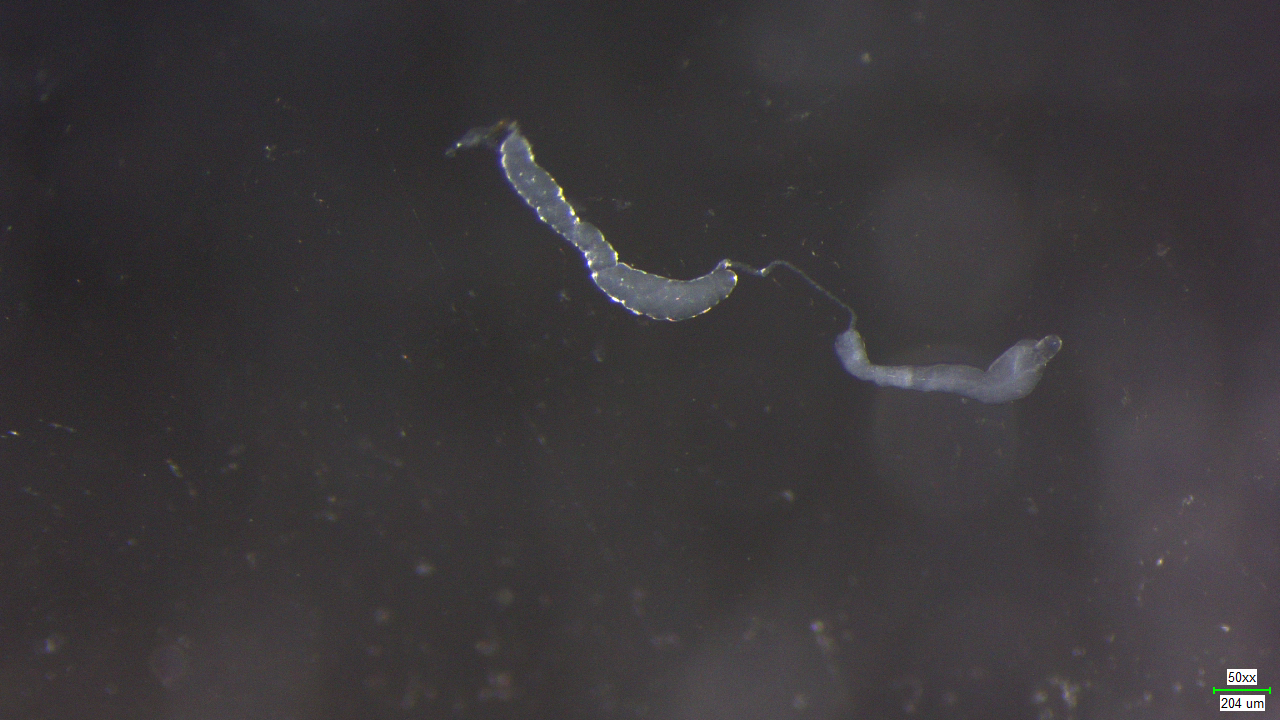

Supplement: Supplementary file 21 — Source data Fig. 4 [file 44318_2026_702_MOESM21_ESM.zip › Figure 4/4D/ds5510.tif]

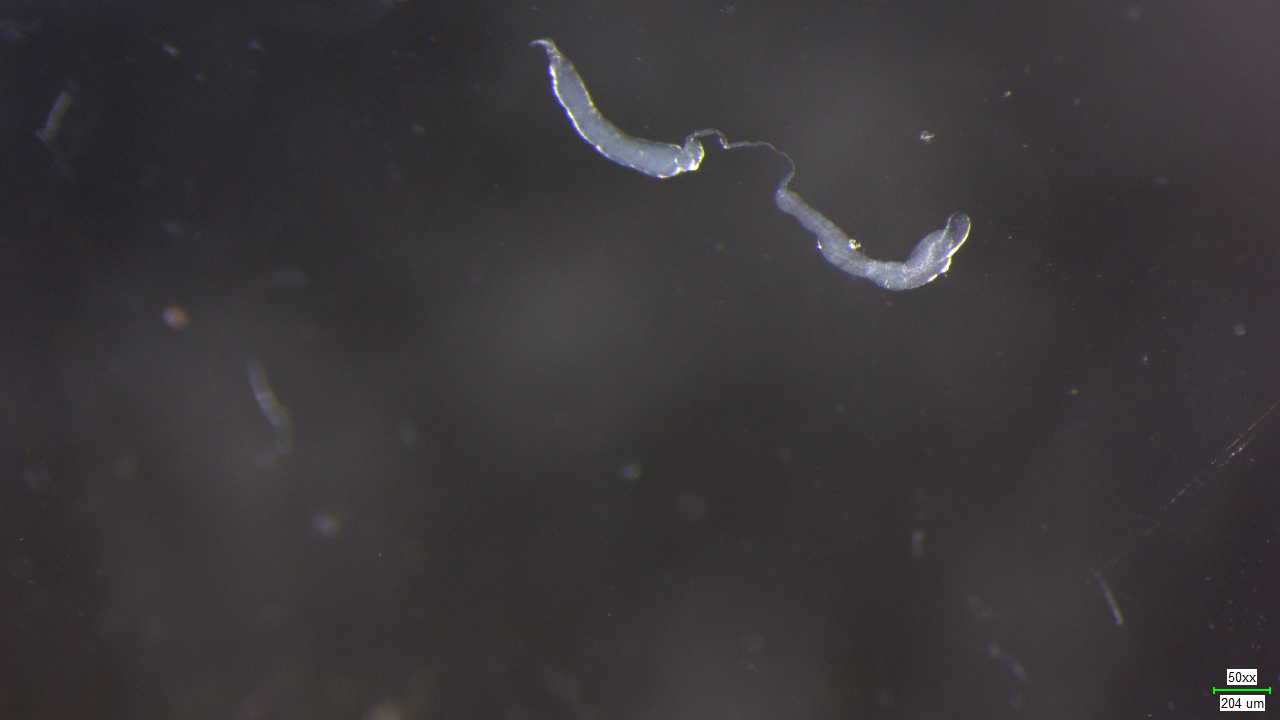

Supplement: Supplementary file 21 — Source data Fig. 4 [file 44318_2026_702_MOESM21_ESM.zip › Figure 4/4D/dsSec24AB.tif]

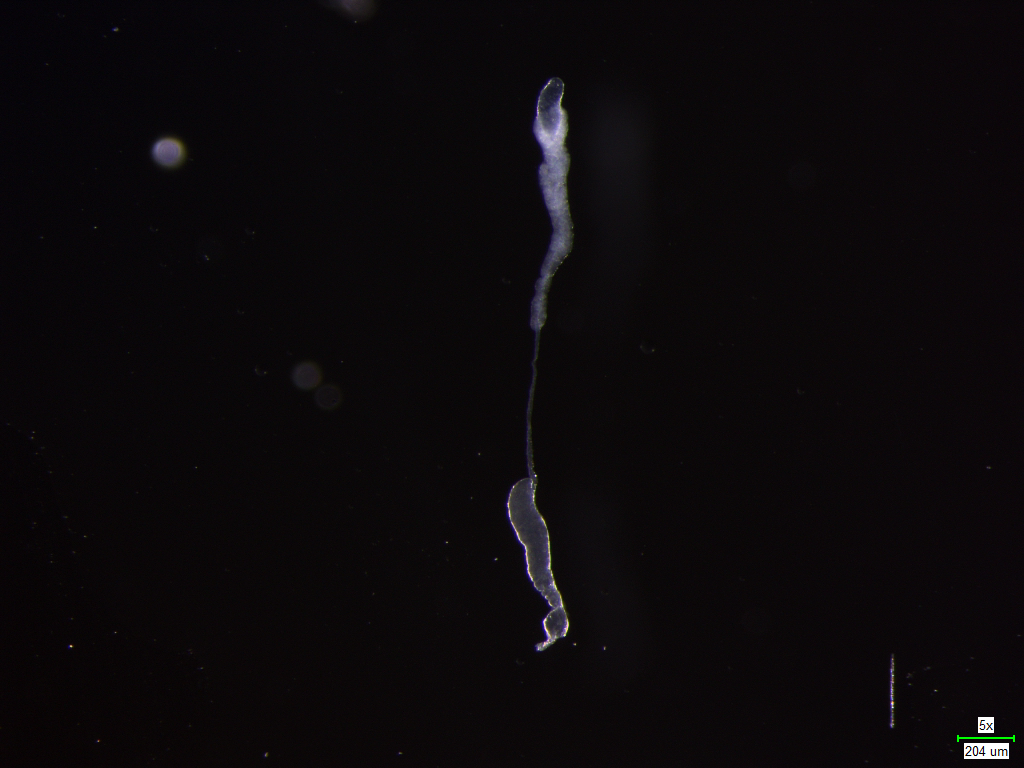

Supplement: Supplementary file 21 — Source data Fig. 4 [file 44318_2026_702_MOESM21_ESM.zip › Figure 4/4D/dsAP-1╬╝.tif]

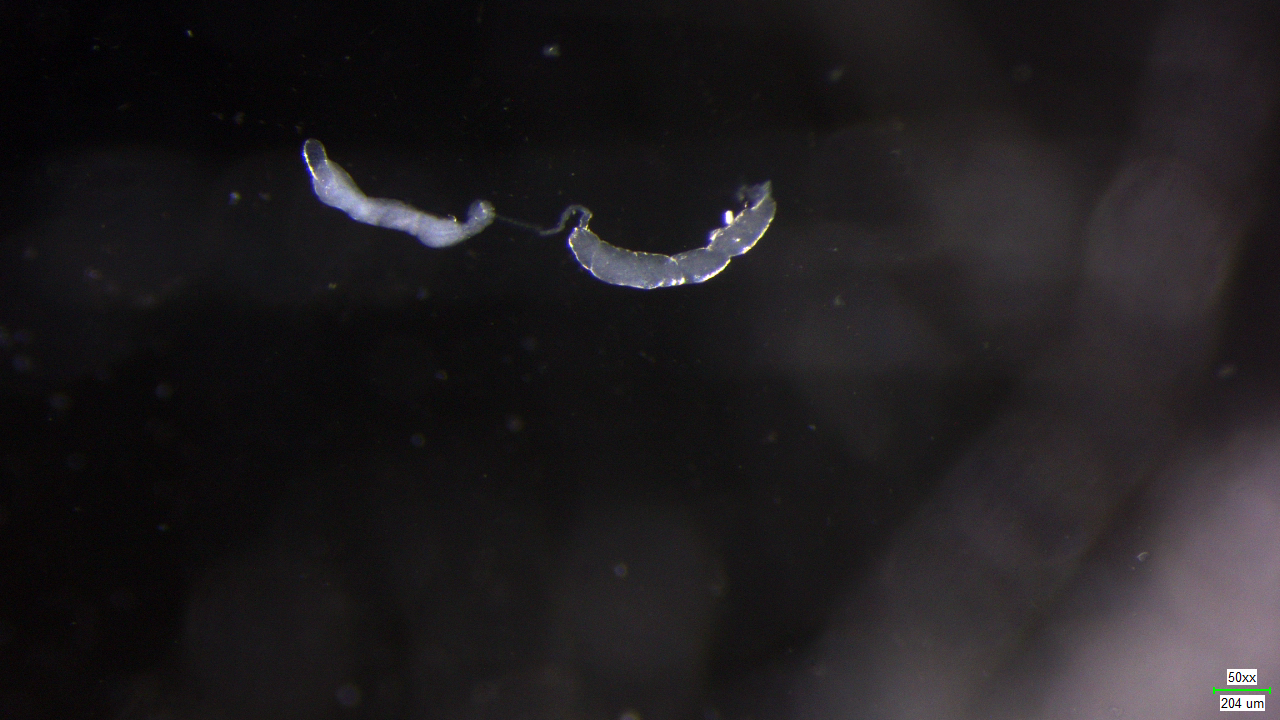

Supplement: Supplementary file 21 — Source data Fig. 4 [file 44318_2026_702_MOESM21_ESM.zip › Figure 4/4D/dsVPS53.tif]

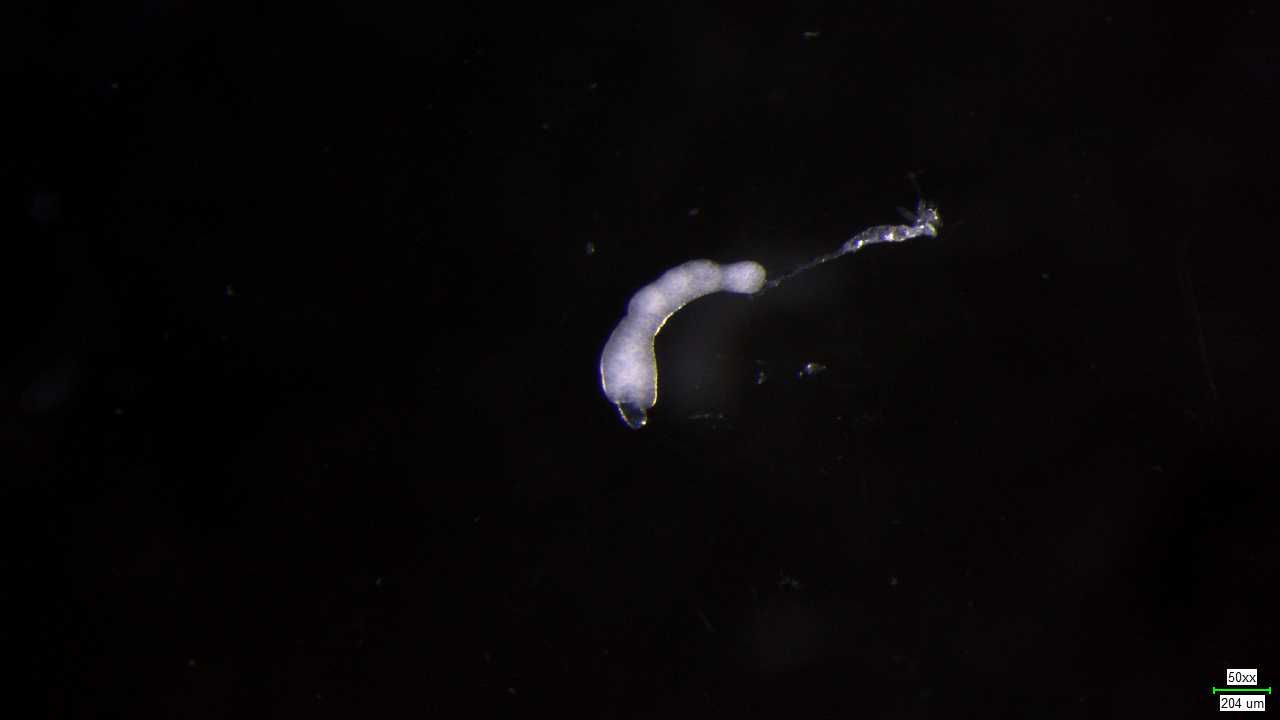

Supplement: Supplementary file 21 — Source data Fig. 4 [file 44318_2026_702_MOESM21_ESM.zip › Figure 4/4D/ds1116.tif]

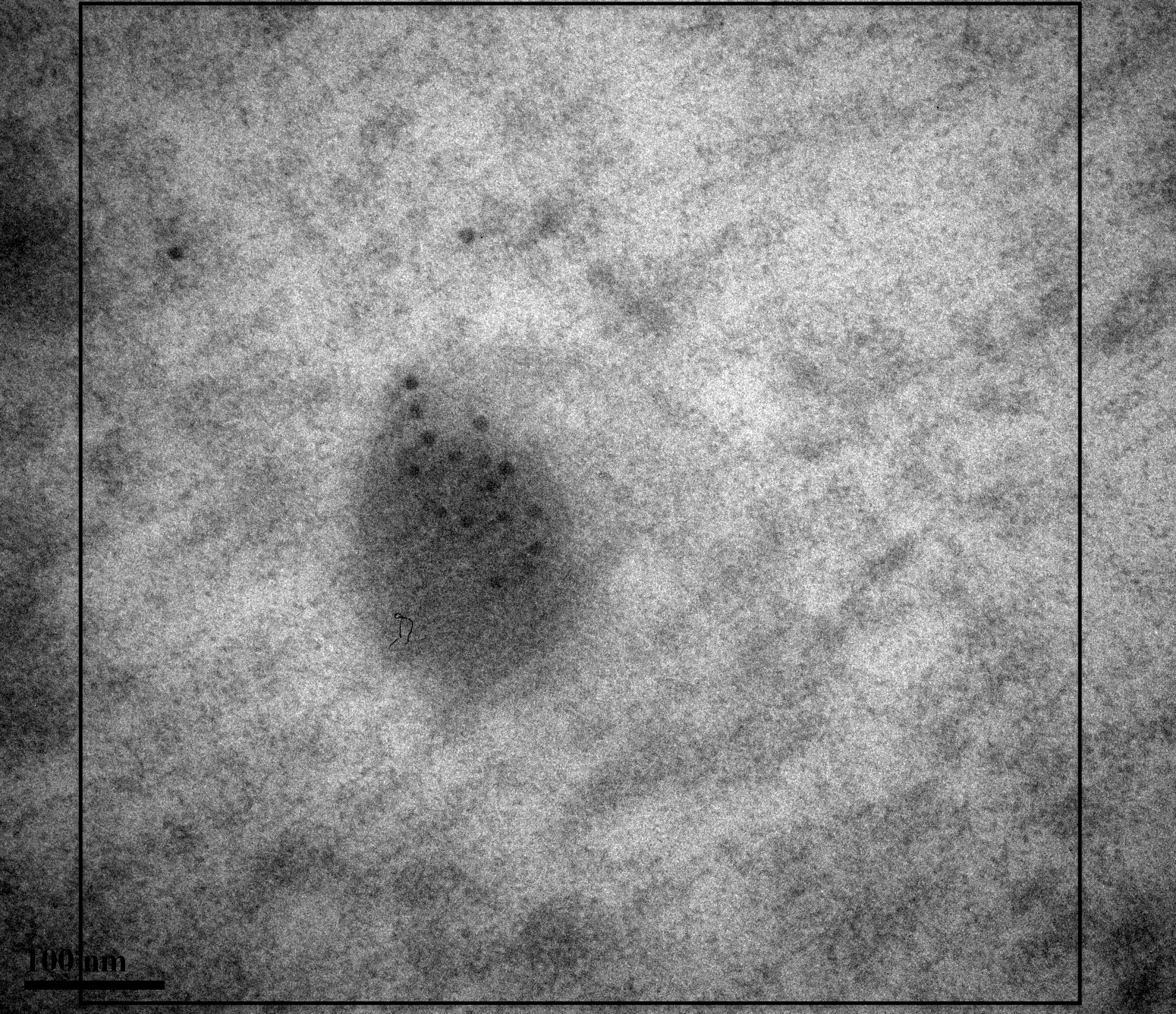

Supplement: Supplementary file 22 — Source data Fig. 5 [file 44318_2026_702_MOESM22_ESM.zip › Figure 5/Figure 5/intracellular vesicle-Anti Lar.tif]

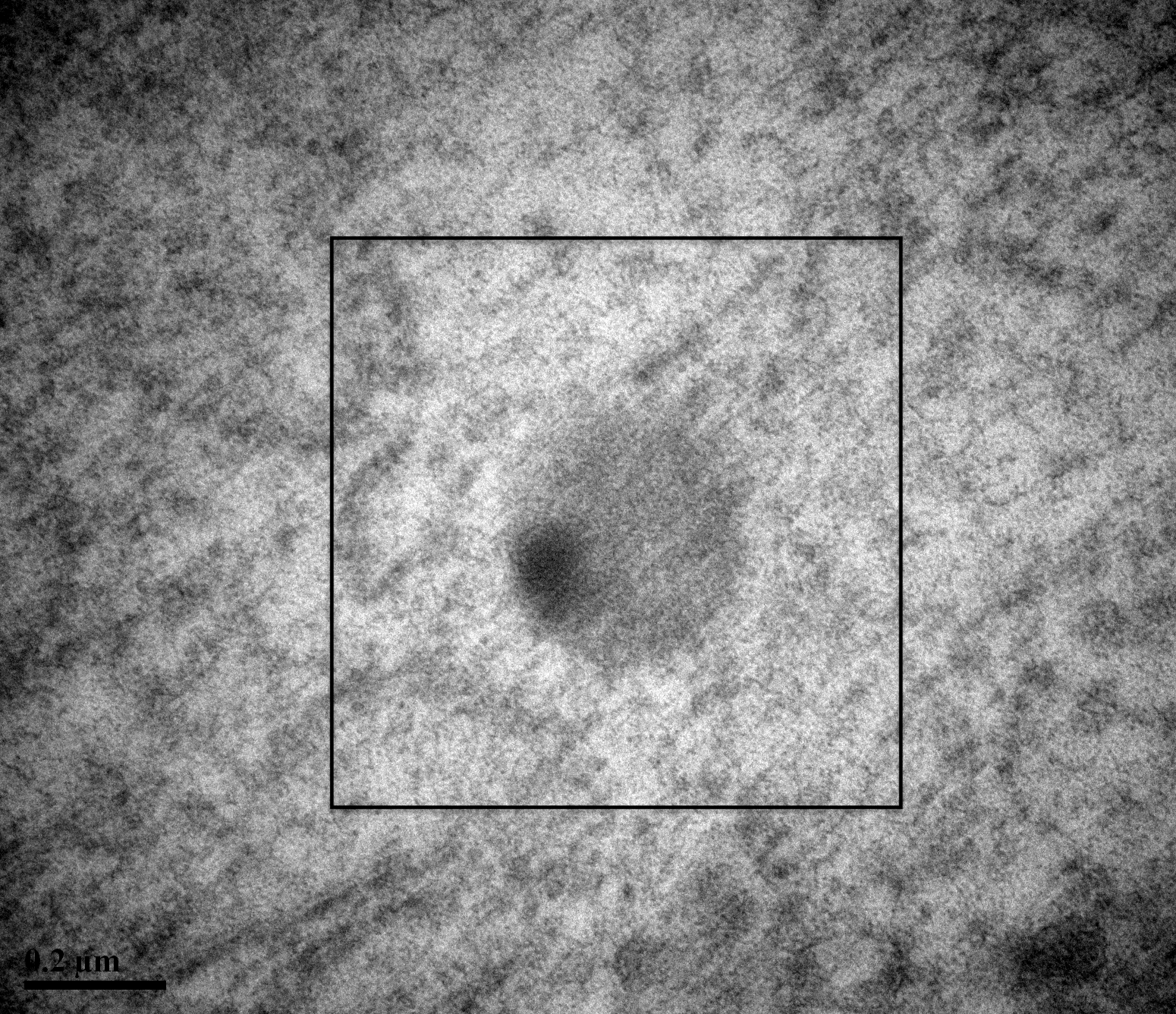

Supplement: Supplementary file 22 — Source data Fig. 5 [file 44318_2026_702_MOESM22_ESM.zip › Figure 5/Figure 5/intracellular vesicle-Negative control.tif]

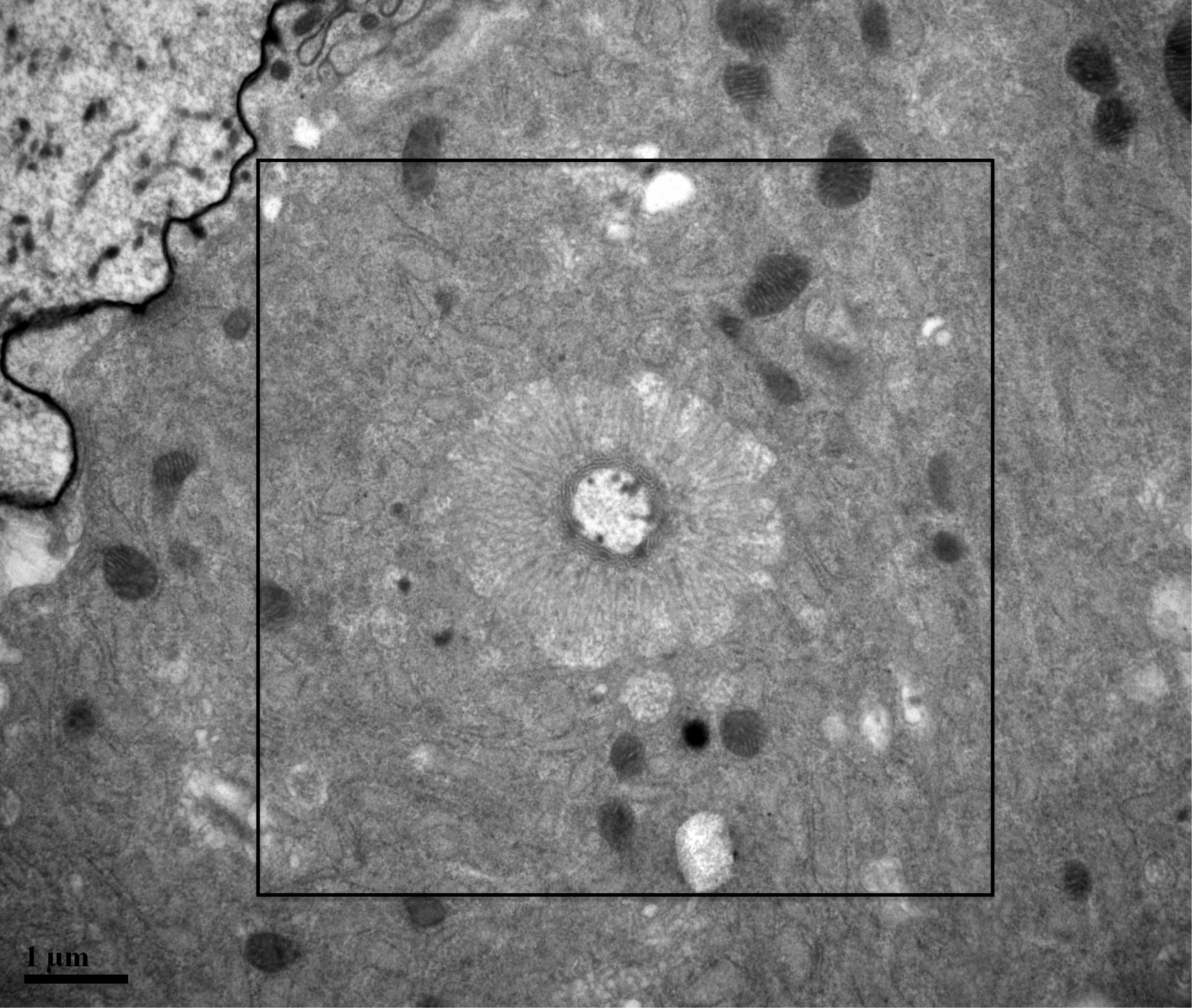

Supplement: Supplementary file 22 — Source data Fig. 5 [file 44318_2026_702_MOESM22_ESM.zip › Figure 5/Figure 5/mixed-strategy extracellular vesicles-dsPTCDS.tif]

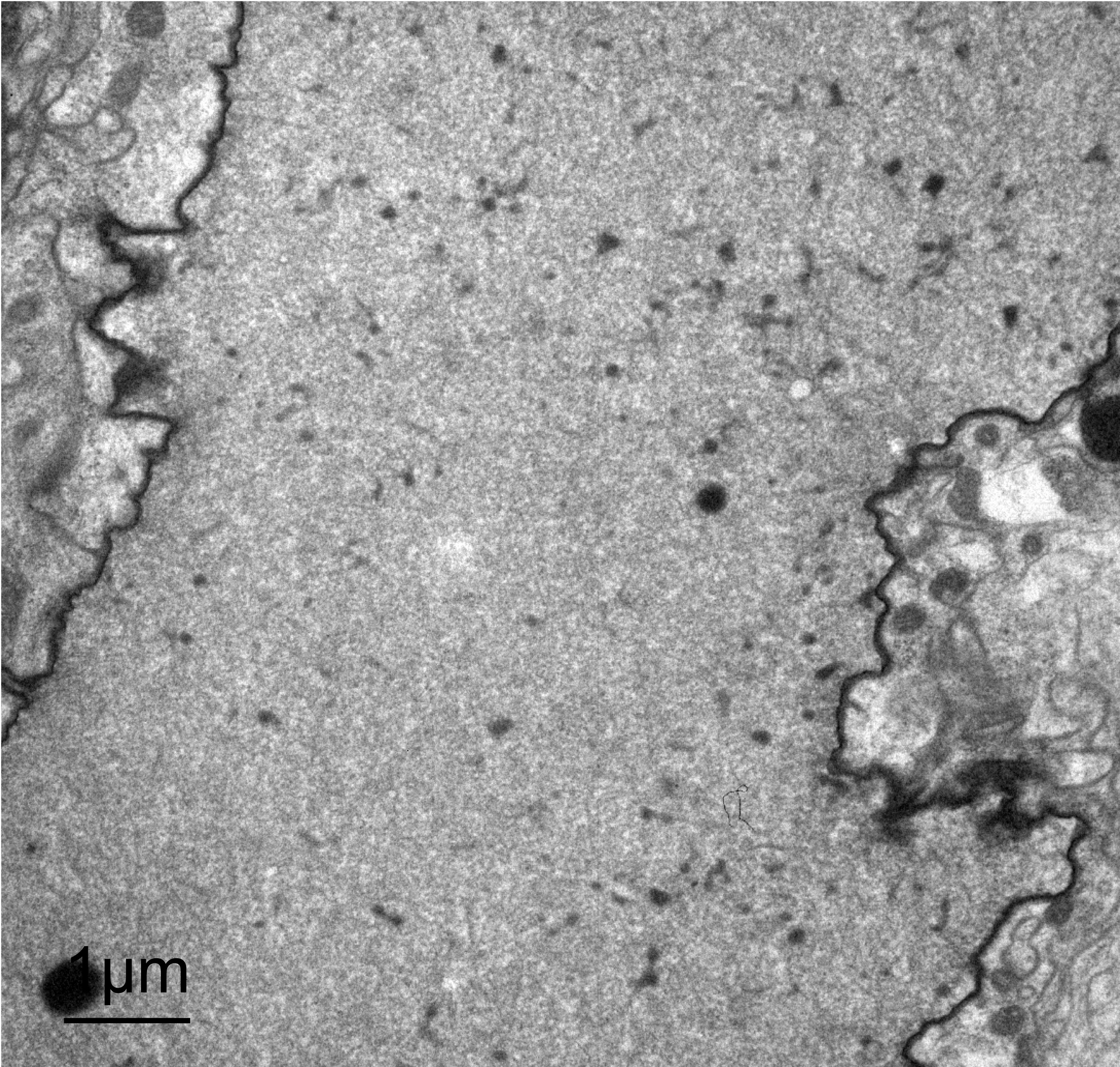

Supplement: Supplementary file 22 — Source data Fig. 5 [file 44318_2026_702_MOESM22_ESM.zip › Figure 5/Figure 5/venom gland lumen-dsPTCDS.tif]

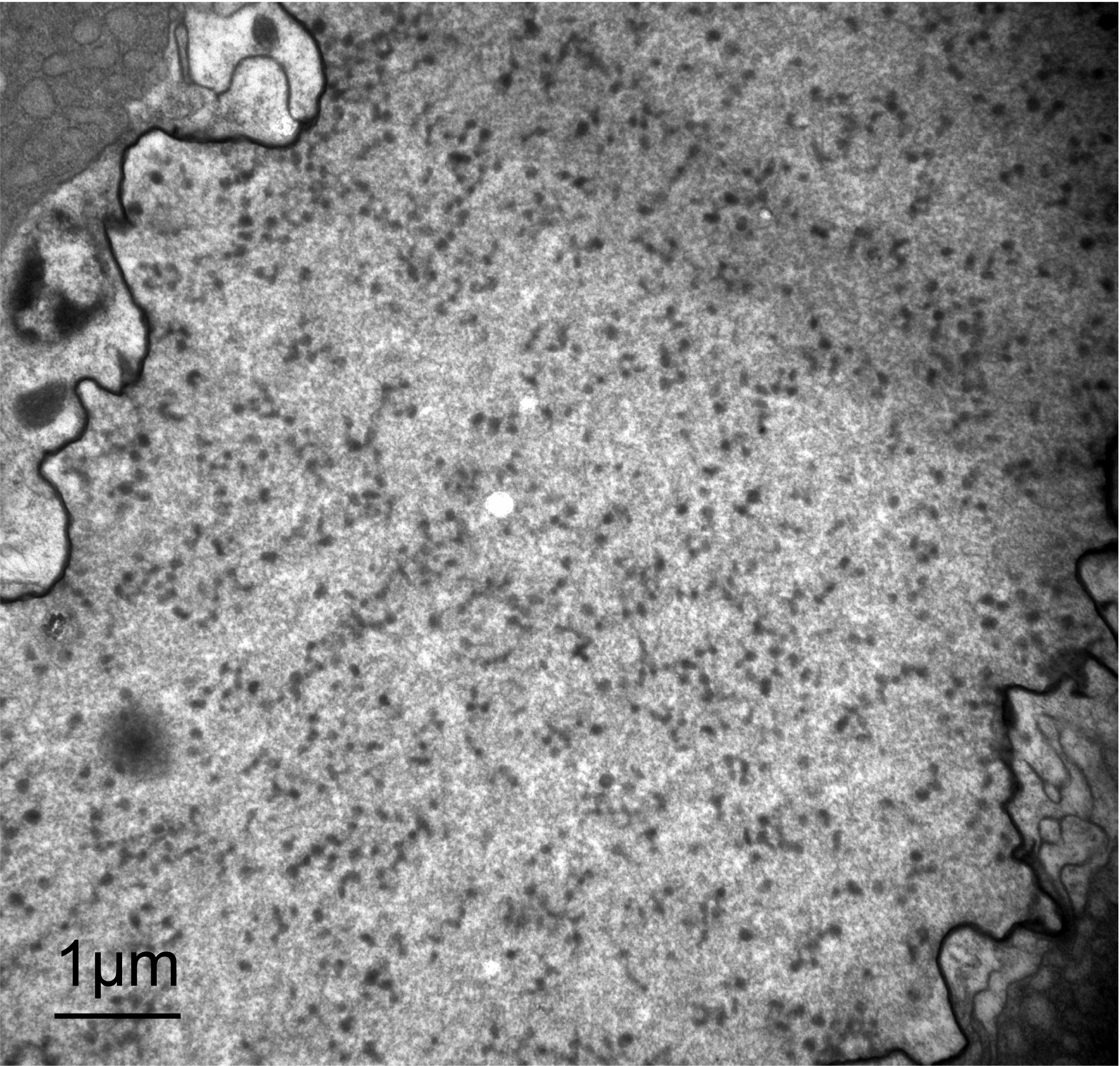

Supplement: Supplementary file 22 — Source data Fig. 5 [file 44318_2026_702_MOESM22_ESM.zip › Figure 5/Figure 5/venom gland lumen-dsGFP.tif]

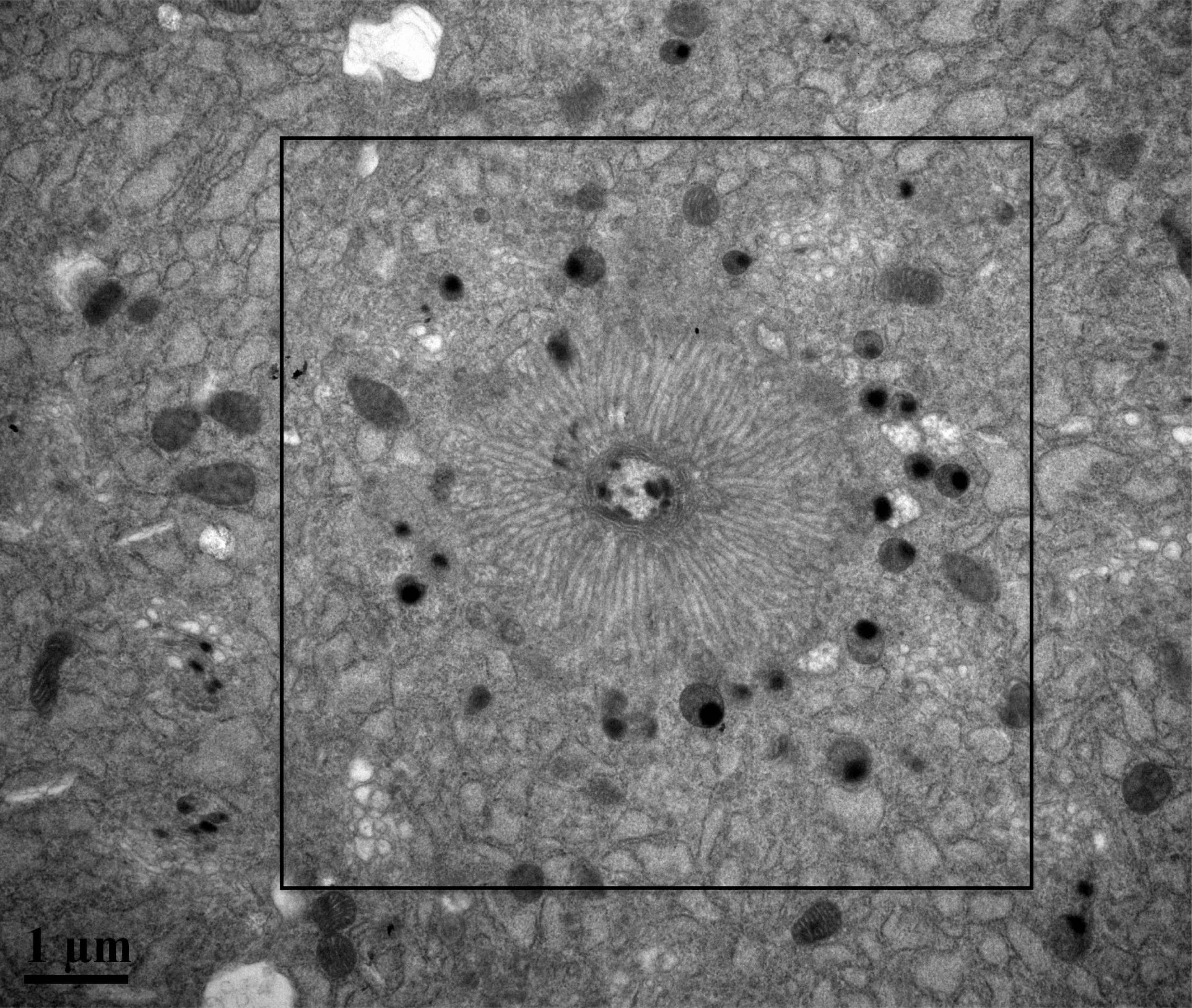

Supplement: Supplementary file 22 — Source data Fig. 5 [file 44318_2026_702_MOESM22_ESM.zip › Figure 5/Figure 5/mixed-strategy extracellular vesicles-dsGFP.tif]
