# Supplementary material for: A bacterial gene acquired by parasitoid wasps contributes to venom secretion against host defence
Source: EMBO J. 2026 Jan 28;45(6):1997–2029. doi: 10.1038/s44318-026-00702-6 (PMC12992598; doi:10.1038/s44318-026-00702-6)
Supplement: Supplementary file 29 — Expanded View Figures [file 44318_2026_702_MOESM29_ESM.pdf]

Expanded View Figures

Figure EV1. A phylogenetic tree of a gene family with putative HGT event3.

The phylogeny was reconstructed (using a 90% identity-filtered sequence set) using IQ-TREE under the best-fit model selected by the -m MFP option. The resulting tree was midpoint-rooted, and branch support is shown as ultrafast bootstrap values (only values <95% are indicated near the internodes). Red branches indicate *L. heterotoma* and *L. syphax*, while cyan branches indicate Bacteria.

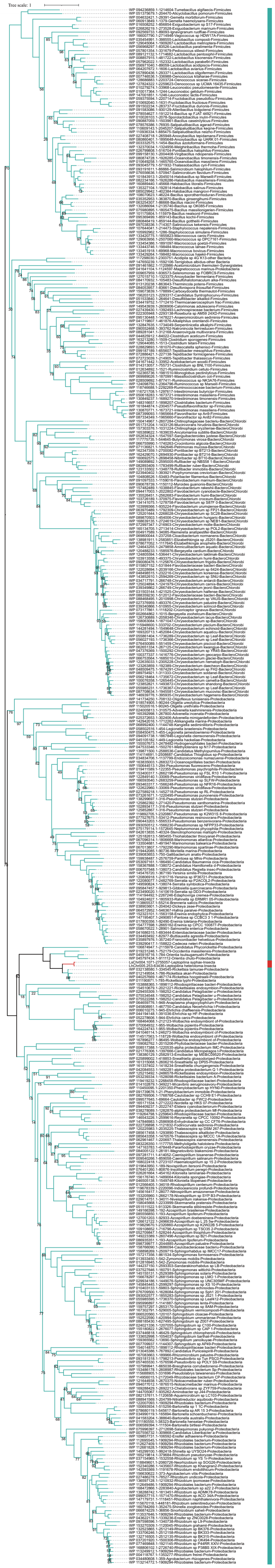

© The Author(s)

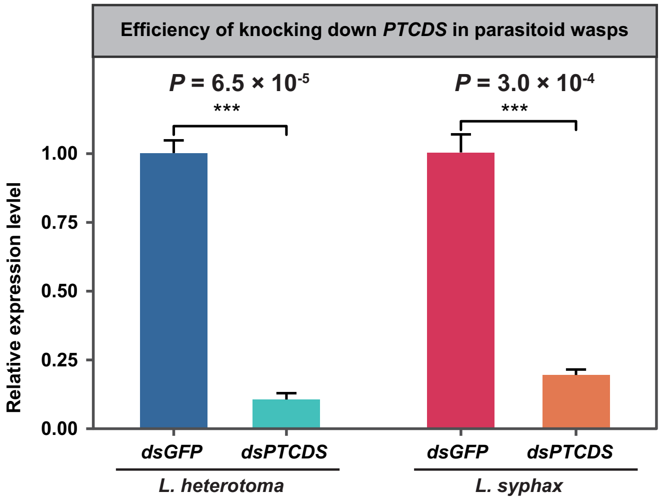

Figure EV2. Efficiency of knocking down *PTCDS* in parasitoid wasps *L. heterotoma* and *L. syphax*.

dsGFP was used as a control. Whole body of 3-day-old female adult *L. heterotoma* and 9-day-old female adult *L. syphax* was used to examine the gene repression. Three replicates were performed for each treatment. Data are presented as mean ± SEM. Significance was determined by two-tailed Student's *t* test (***P < 0.001). Source data are available online for this figure.

© The Author(s)

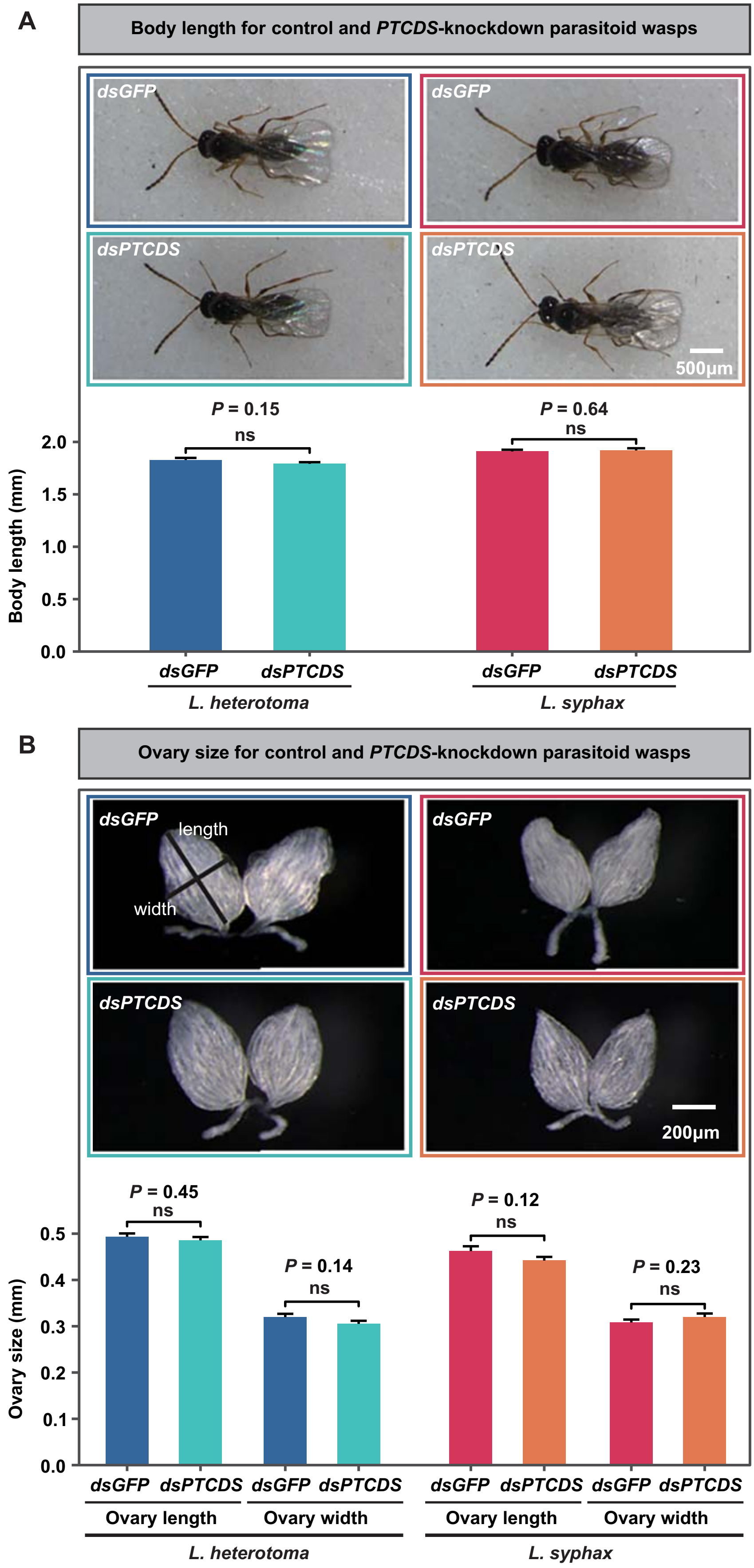
A
Body length for control and PTCDS-knockdown parasitoid wasps
dsGFP
dsGFP
dsPTCDS
dsPTCDS
500µm
P = 0.15
ns
P = 0.64
ns
Body length (mm)
2.0
1.5
1.0
0.5
0.0
dsGFP
dsPTCDS
L. heterotoma
dsGFP
dsPTCDS
L. syphax
B
Ovary size for control and PTCDS-knockdown parasitoid wasps
dsGFP
length
width
dsGFP
dsPTCDS
dsPTCDS
200µm
P = 0.45
ns
P = 0.14
ns
P = 0.12
ns
P = 0.23
ns
Ovary size (mm)
0.5
0.4
0.3
0.2
0.1
0.0
dsGFP dsPTCDS dsGFP dsPTCDS dsGFP dsPTCDS dsGFP dsPTCDS
Ovary length
Ovary width
Ovary length
Ovary width
L. heterotoma
L. syphax

 © The Author(s)

Figure EV3. Comparison of developmental phenotypes between control and *PTCDS*-knockdown parasitoid wasps.

3-day-old female adult *L. heterotoma* ($n = 30$) and 9-day-old female adult *L. syphax* ($n = 25$) were used to examine developmental phenotypes, respectively. *dsGFP* was used as a control. (**A**) Effects of *PTCDS* on parasitoid wasp body length. (**B**) Effects of *PTCDS* on parasitoid wasp ovary size (length and width). Note that the length and width sizes are the average values of the pair of ovaries from each individual. Data are presented as mean ± SEM. Significance was determined by two-tailed Student's *t* test (ns, not significant). Source data are available online for this figure.

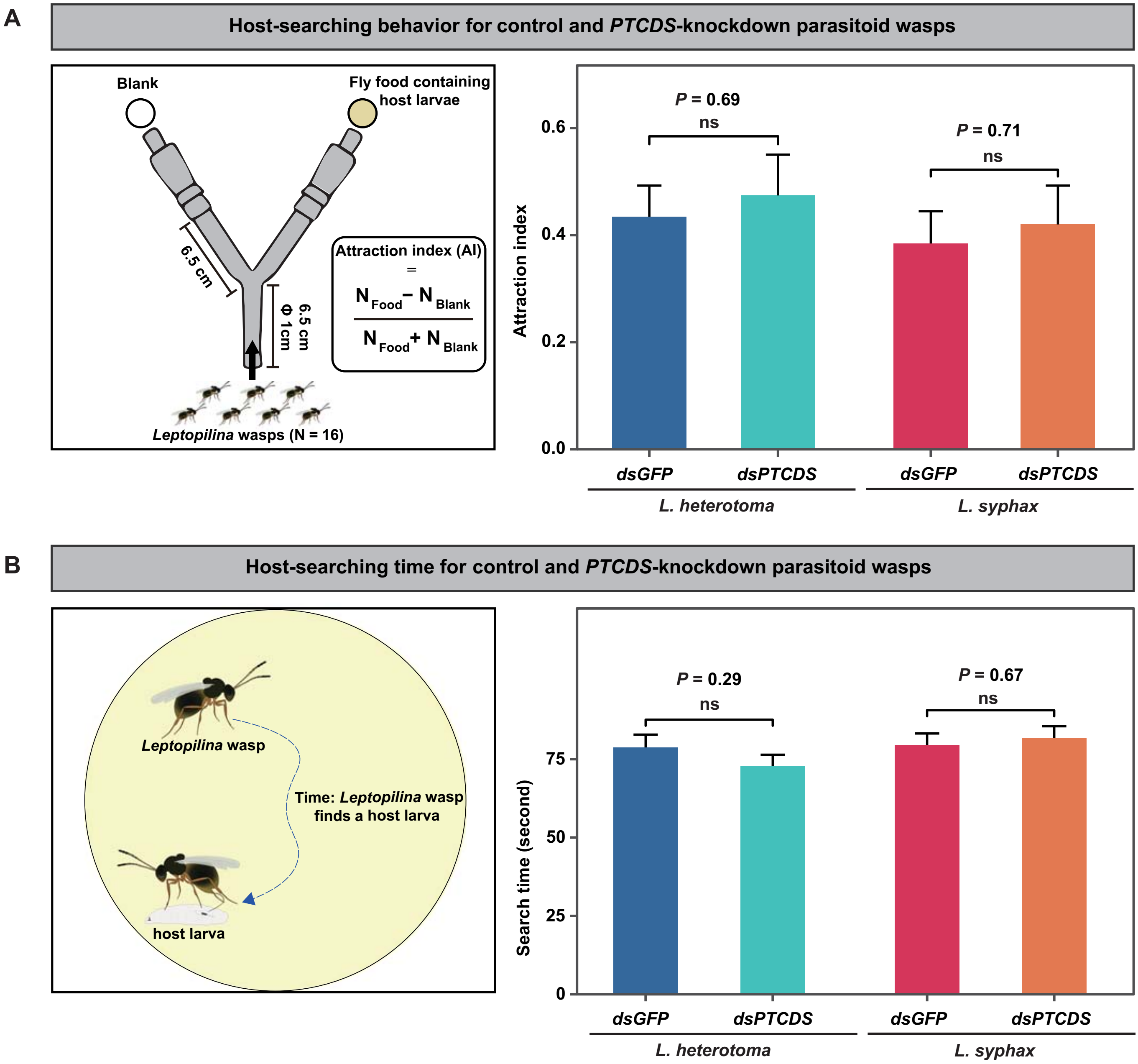

Figure EV4. Comparison of host-searching behaviors for control and *PTCDS*-knockdown parasitoid wasps.

(**A**) Effects of *PTCDS* on host searching ability for parasitoid wasps using Y-tube assay. The left panel is a schematic diagram of Y-tube behavioral assay for monitoring the host-searching behavior (Attraction Index, AI). The right panel is the summary of AI values for control and *PTCDS*-knockdown parasitoid wasps. *dsGFP* was used as a control. Six replicates were performed for each treatment. Data are presented as mean ± SEM. Significance was determined by two-tailed Student's *t* test (ns, not significant). (**B**) Effects of *PTCDS* on the time of searching host larvae for parasitoid wasps. The left panel is a schematic diagram of an assay for monitoring time that a wasp found a host larva. The right panel is the summary of the time of searching host. *dsGFP* was used as a control. Fifteen replicates were performed for each treatment. Data are presented as mean ± SEM. Significance was determined by two-tailed Student's *t* test (ns, not significant). Source data are available online for this figure.

 © The Author(s)

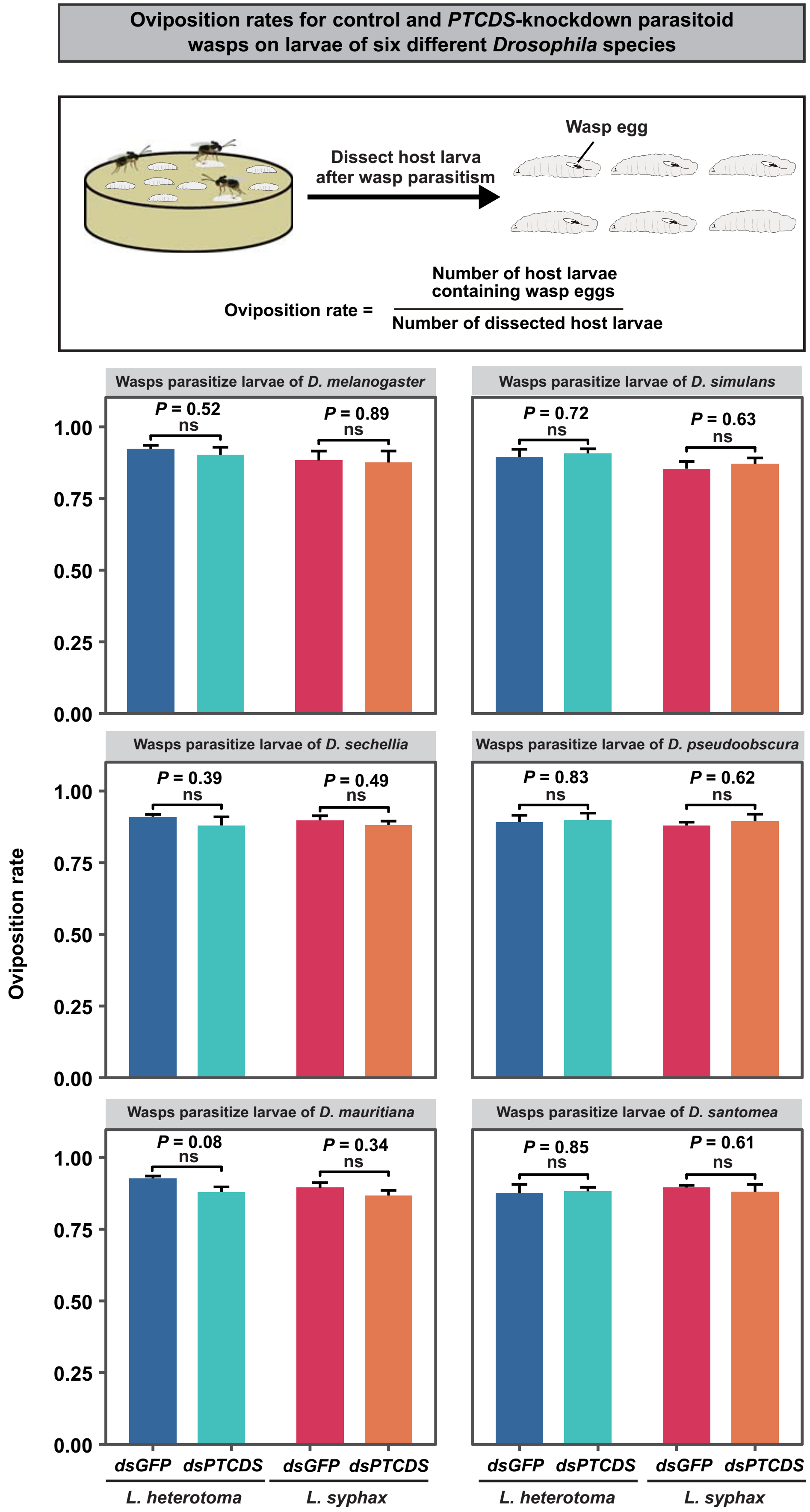
Oviposition rates for control and PTCDS-knockdown parasitoid wasps on larvae of six different Drosophila species
Wasp egg
Dissect host larva after wasp parasitism
Oviposition rate = Number of host larvae containing wasp eggs / Number of dissected host larvae
Wasps parasitize larvae of D. melanogaster
P = 0.52 ns
P = 0.89 ns
Wasps parasitize larvae of D. simulans
P = 0.72 ns
P = 0.63 ns
Wasps parasitize larvae of D. sechellia
P = 0.39 ns
P = 0.49 ns
Wasps parasitize larvae of D. pseudoobscura
P = 0.83 ns
P = 0.62 ns
Wasps parasitize larvae of D. mauritiana
P = 0.08 ns
P = 0.34 ns
Wasps parasitize larvae of D. santomea
P = 0.85 ns
P = 0.61 ns
Oviposition rate
1.00
0.75
0.50
0.25
0.00
dsGFP dsPTCDS dsGFP dsPTCDS
L. heterotoma L. syphax

Figure EV5. Comparison of oviposition rate between control and *PTCDS*-knockdown parasitoid wasps.

The upper panel is a schematic diagram for examining the oviposition rate of parasitoid wasps. The below panel is the summary of oviposition rates of parasitoid wasps that parasitized for each of six different *Drosophila* host larvae. *dsGFP* was used as a control. The ratio of female wasps to host larvae is 3:360 and 3:180 for 3-day-old *L. heterotoma* and 9-day-old *L. syphax*, respectively. Three replicates were performed for each treatment. Data are presented as mean ± SEM. Significance was determined by two-tailed Student's *t* test (ns, not significant). Source data are available online for this figure.

© The Author(s)

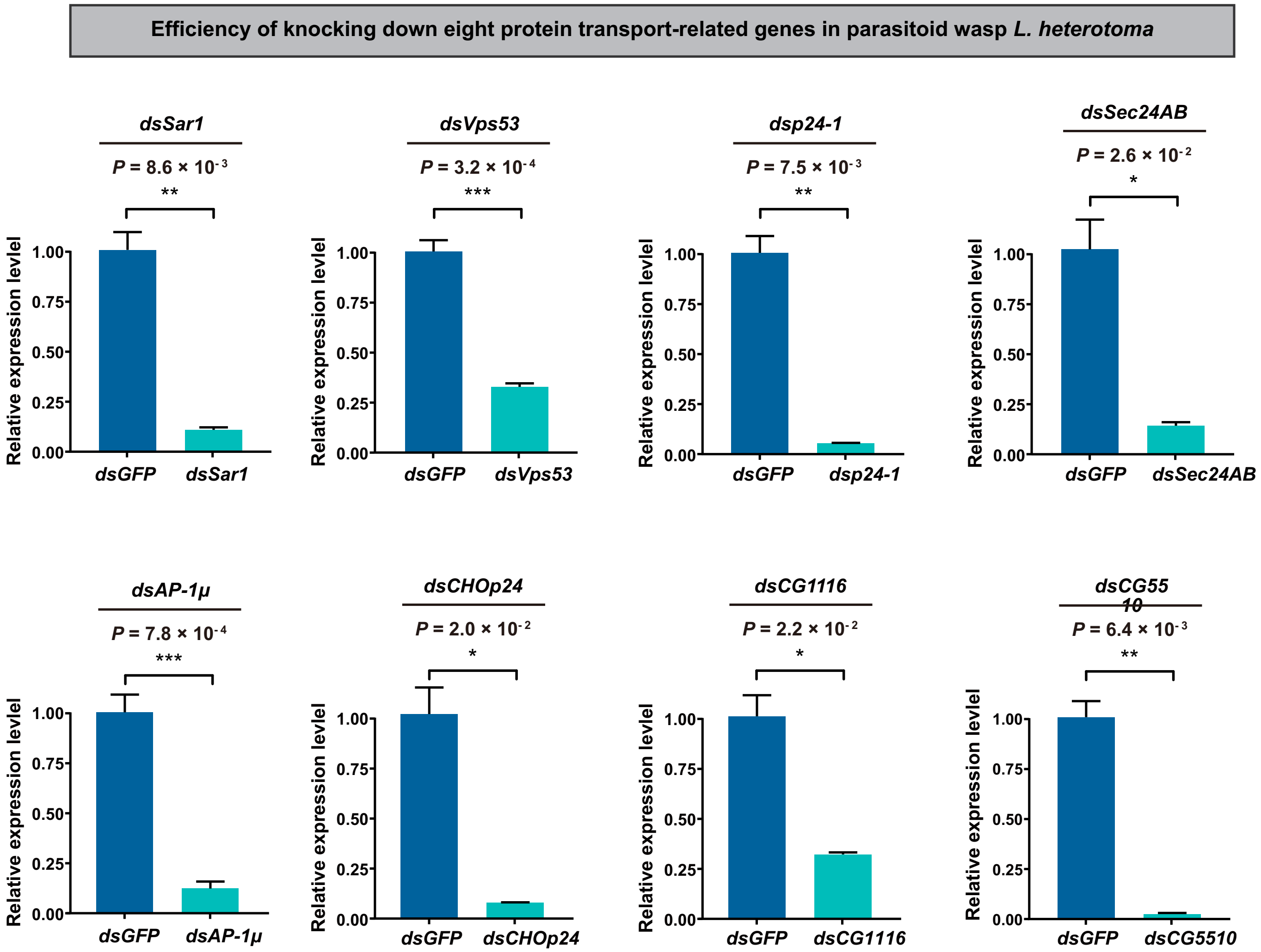

Figure EV6. Efficiency of knocking down eight protein transport-related genes in parasitoid wasp *L. heterotoma*.

dsGFP was used as a control. Whole body of 3-day-old female adult *L. heterotoma* was used to examine the gene repression. Three replicates were performed for each treatment. Data are presented as mean ± SEM. Statistical analysis was performed using two-tailed Student's *t* test when parametric assumptions and homogeneity of variances were met, and Welch's *t* test was used to determine significance when parametric assumptions were met but heterogeneity of variances was observed ($^*P < 0.05$; $^{**}P < 0.01$; $^{***}P < 0.001$). Source data are available online for this figure.

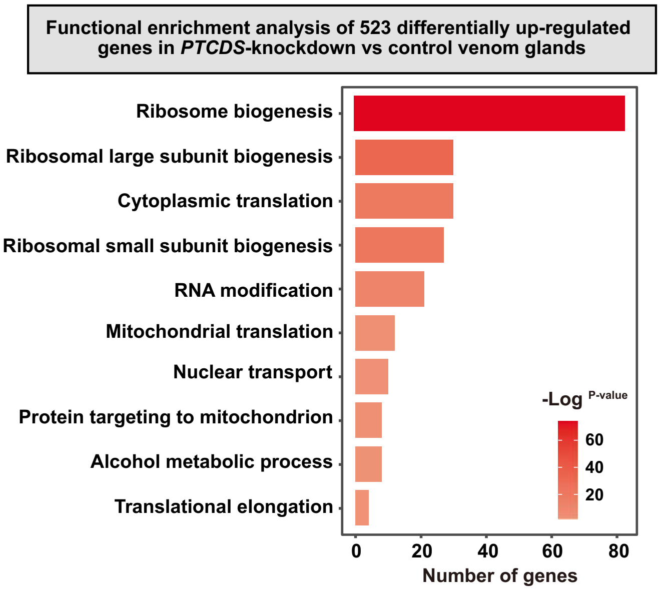

Figure EV7. Functional enrichment analysis of 523 differentially upregulated genes.

We conducted functional enrichment analysis of 523 differentially upregulated genes between *PTCDS*-knockdown and control venom glands of 3-day-old female adult *L. heterotoma*. Statistical significance was assessed using the hypergeometric test, *P* value < 0.01.

 © The Author(s)

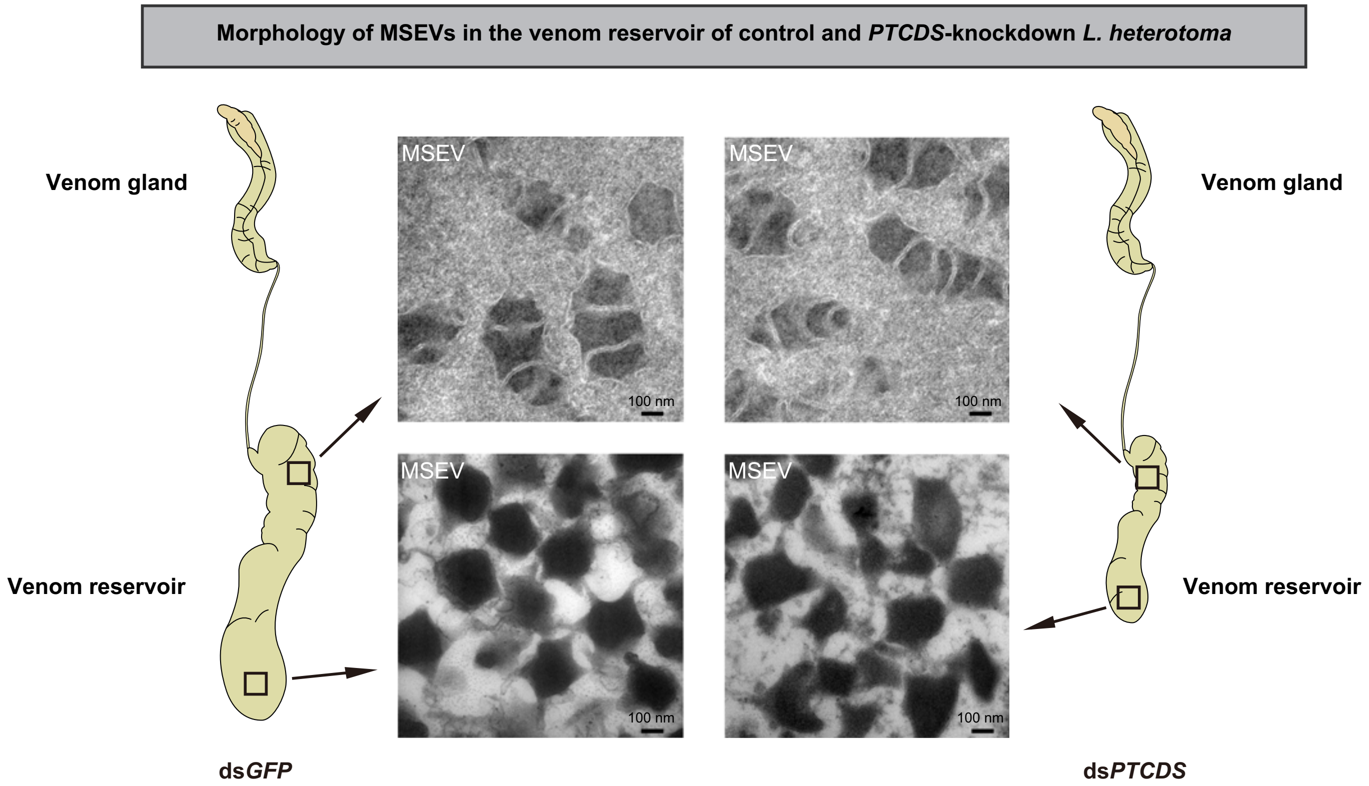

Figure EV8. ***PTCDS* knockdown in *L. heterotoma* did not alter MSEV morphology.**

Comparison of transmission electron micrographs (TEMs) of MSEV morphology in the anterior and posterior regions of venom reservoirs from the control and *PTCDS*-knockdown 3-day-old female adult *L. heterotoma*.

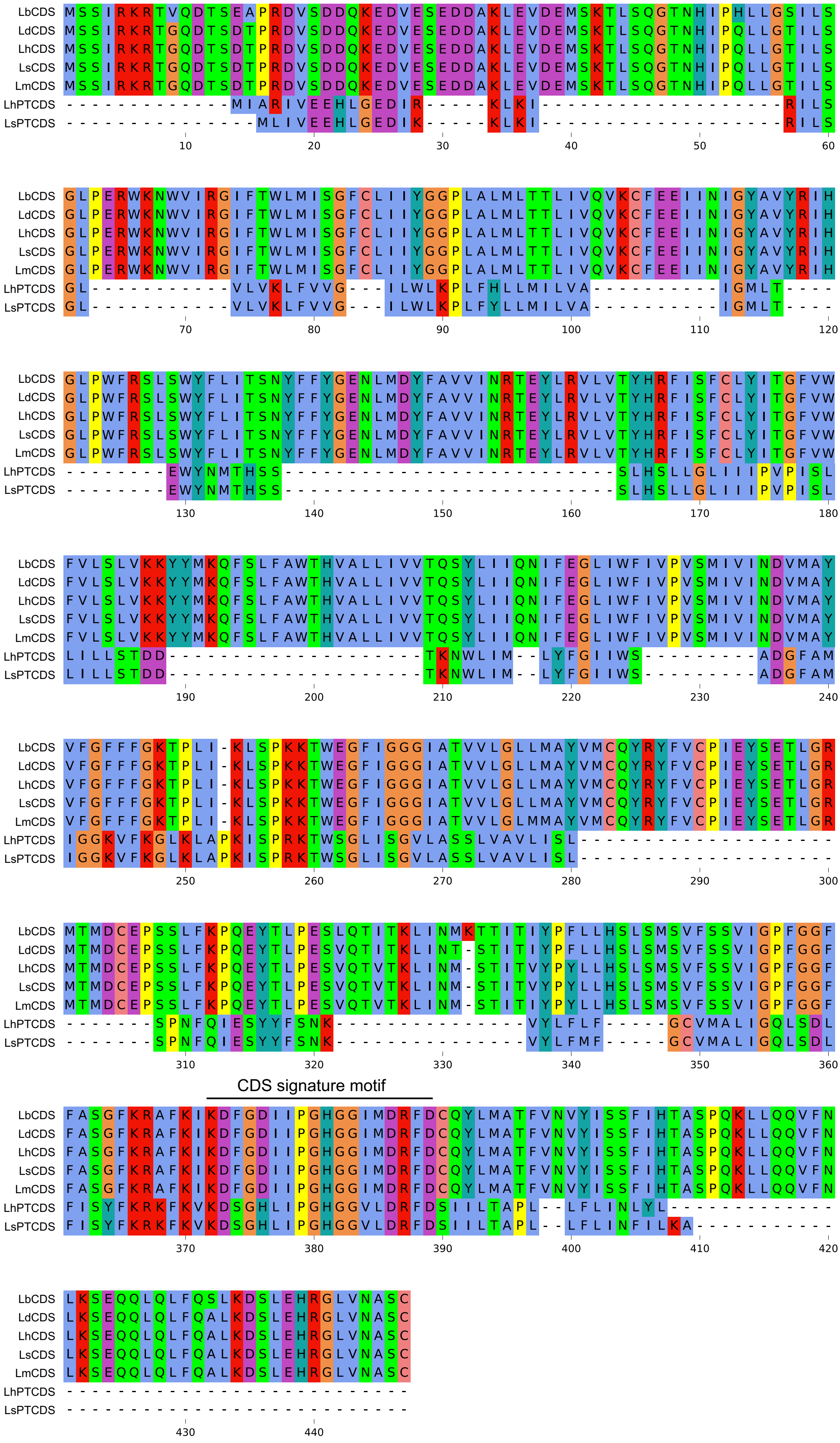
LbCDS
LdCDS
LhCDS
LsCDS
LmCDS
LhPTCDS
LsPTCDS
CDS signature motif

© The Author(s)

Figure EV9. Comparison of eukaryotic-type and prokaryotic-type CDS protein sequences.

The eukaryotic-type CDS proteins of *L. boulardi* (LbCDS), *L. drosophilae* (LdCDS), *L. heterotoma* (LhCDS), *L. syphax* (LsCDS) and *L. myrica* (LmCDS) exhibited high sequence conservation, sharing 97% amino acid identity. Although the eukaryotic-type CDS shares only ~27% amino acid identity with the PTCDS in *L. heterotoma* and *L. syphax*, all homologs contain a CDP-diacylglycerol synthetase domain, including a conserved CDS signature motif (marked by a black line).

© The Author(s)

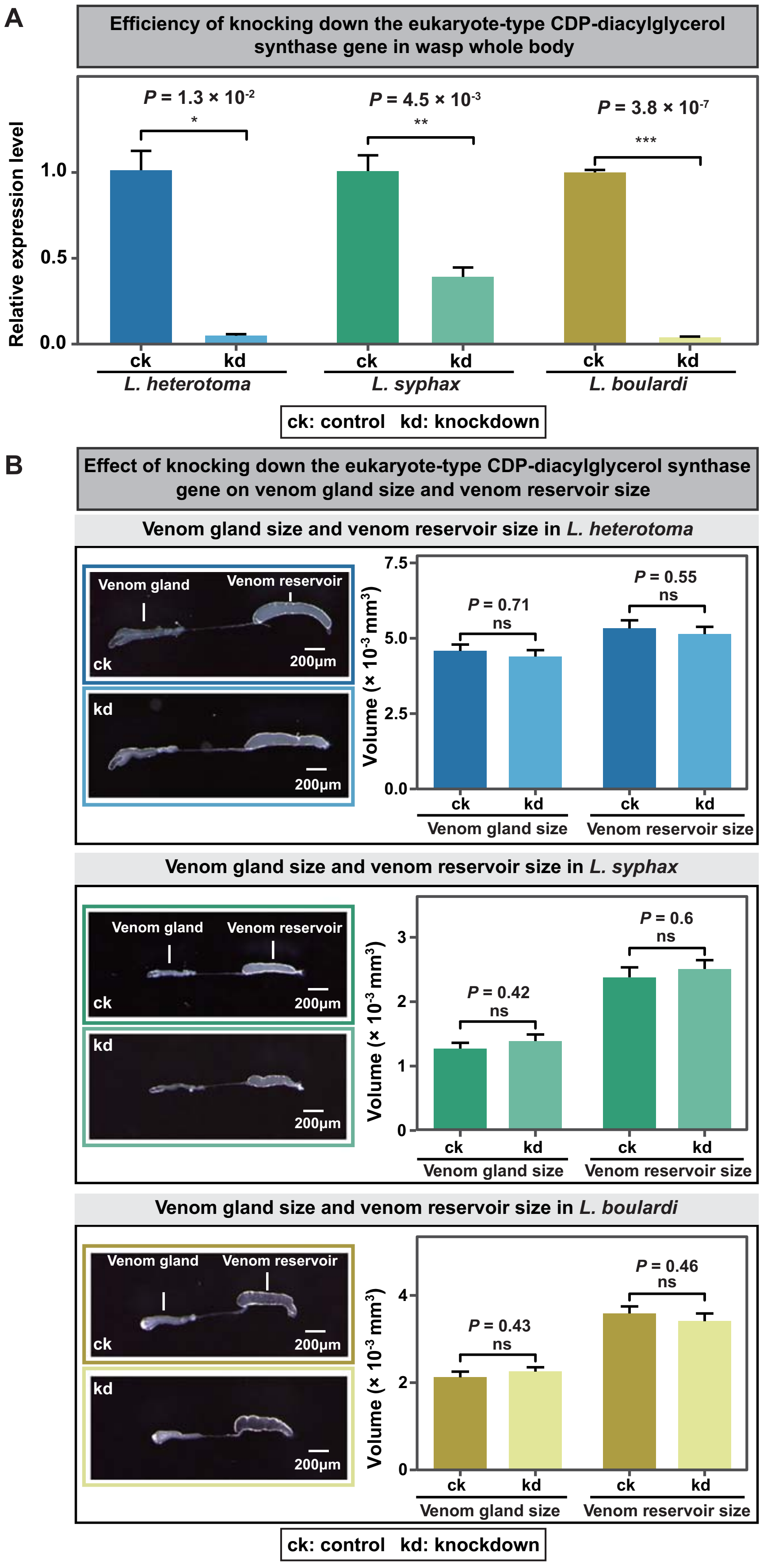
A
Efficiency of knocking down the eukaryote-type CDP-diacylglycerol synthase gene in wasp whole body
Relative expression level
P = 1.3 × 10-2
*
P = 4.5 × 10-3
**
P = 3.8 × 10-7
***
ck kd ck kd ck kd
L. heterotoma
L. syphax
L. boulardi
ck: control kd: knockdown
B
Effect of knocking down the eukaryote-type CDP-diacylglycerol synthase gene on venom gland size and venom reservoir size
Venom gland size and venom reservoir size in L. heterotoma
Venom gland
Venom reservoir
ck
kd
200μm
Volume (× 10-3 mm3)
P = 0.71
ns
P = 0.55
ns
Venom gland size
Venom reservoir size
Venom gland size and venom reservoir size in L. syphax
P = 0.42
ns
P = 0.6
ns
Venom gland size and venom reservoir size in L. boulardi
P = 0.43
ns
P = 0.46
ns
ck: control kd: knockdown

 © The Author(s)

Figure EV10. Effects of the eukaryote-type CDP-diacylglycerol synthase gene on venom gland size and venom reservoir size of parasitoid wasps.

Although *L. heterotoma* and *L. syphax* horizontally acquired the prokaryote-type CDP-diacylglycerol synthase gene (*PTCDS*) from the bacterial family Rickettsiaceae, we found that all 5 parasitoid wasps (*L. heterotoma*, *L. syphax*, *L. drosophilae*, *L. boulardi*, and *L. myrica*) contain the eukaryote-type CDP-diacylglycerol synthase gene. The eukaryote-type CDP-diacylglycerol synthase gene shares ~20% amino acid identity with the prokaryote-type CDP-diacylglycerol synthase gene (*PTCDS*) in *L. heterotoma* and *L. syphax*. To examine the effects of eukaryote-type CDP-diacylglycerol synthase gene on venom system, we used RNAi to knock down the expression level of the eukaryote-type CDP-diacylglycerol synthase gene in *L. heterotoma, L. syphax*, and *L. boulardi*, respectively. (**A**) Efficiency of knocking down eukaryote-type CDP-diacylglycerol synthase gene in *L. heterotoma, L. syphax*, and *L. boulardi*. Three replicates were performed for each treatment. Data are presented as mean ± SEM. Statistical analysis was performed using two-tailed Student's *t* test when parametric assumptions and homogeneity of variances were met, and Welch's *t* test was used to determine significance when parametric assumptions were met but heterogeneity of variances was observed ($^{*}P < 0.05$; $^{**}P < 0.01$; $^{***}P < 0.001$). (**B**) Effects of knocking down the eukaryote-type CDP-diacylglycerol synthase gene on venom gland size and venom reservoir size in *L. heterotoma, L. syphax*, and *L. boulardi*. Female adult wasps *L. heterotoma* ($n = 30$), *L. syphax* ($n = 23$), and *L. boulardi* ($n = 30$) were used to examine their venom gland sizes and venom reservoir sizes, respectively. Data are presented as mean ± SEM. Statistical analysis was performed using two-tailed Student's *t* test when parametric assumptions and homogeneity of variances were met, Welch's *t* test was used to determine significance when parametric assumptions were met but heterogeneity of variances was observed, and the Mann–Whitney *U* test was used to determine significance when experiments required nonparametric statistical tests (ns, not significant). Source data are available online for this figure.
